# Development of multi epitope subunit vaccines against emerging carp viruses *Cyprinid herpesvirus* 1 and 3 using immunoinformatics approach

**DOI:** 10.1038/s41598-024-61074-7

**Published:** 2024-05-23

**Authors:** Nurul Amin Rani, Tanjin Barketullah Robin, Anindita Ash Prome, Nadim Ahmed, Abu Tayab Moin, Rajesh B. Patil, Mohammad Nurul Azim Sikder, Md Nazmul Islam Bappy, Dilruba Afrin, Ferdaus Mohd Altaf Hossain, Tofazzal Islam, Kazi Md. Ali Zinnah

**Affiliations:** 1 Faculty of Biotechnology and Genetic Engineering, Sylhet Agricultural University, Sylhet, 3100 Bangladesh; 2 Department of Genetic Engineering and Biotechnology, Faculty of Biological Sciences, University of Chittagong, Chattogram, 4331 Bangladesh; 3 Department of Pharmaceutical Chemistry, Sinhgad College of Pharmacy, Sinhgad Technical Education Society’s, Off Sinhgad Road, Vadgaon (Bk), Pune, Maharashtra 411041 India; 4 Institute of Marine Sciences, Faculty of Marine Sciences and Fisheries, University of Chittagong, Chattogram, 4331 Bangladesh; 5 Department of Animal and Fish Biotechnology, Sylhet Agricultural University, Sylhet, 3100 Bangladesh; 6 Faculty of Veterinary, Animal and Biomedical Science, Sylhet Agricultural University, Sylhet, Bangladesh; 7 Department of Dairy Science, Sylhet Agricultural University, Sylhet, Bangladesh; 8 Institute of Biotechnology and Genetic Engineering (IBGE), Bangabandhu Sheikh Mujibur Rahman Agricultural University (BSMRAU), Gazipur, 1706 Bangladesh

**Keywords:** Computational biology and bioinformatics, Drug discovery, Immunology, Molecular biology

## Abstract

*Cyprinid herpesvirus* is a causative agent of a destructive disease in common and koi carp (*Cyprinus carpio*), which leads to substantial global financial losses in aquaculture industries. Among the strains of *C. herpesvirus*, *C. herpesvirus* 1 (CyHV-1) and *C. herpesvirus* 3 (CyHV-3) are known as highly pathogenic to carp fishes in Europe, Asia, and Africa. To date, no effective vaccine has been developed to combat these viruses. This study aimed to develop unique multi-epitope subunit vaccines targeting the CyHV-1 and CyHV-3 using a reverse vaccinology approach. The study began with a comprehensive literature review to identify the most critical proteins, which were then subjected to in silico analyses to predict highly antigenic epitopes. These analyses involved assessing antigenicity, transmembrane topology screening, allergenecity, toxicity, and molecular docking approaches. We constructed two multi-epitope-based vaccines incorporating a suitable adjuvant and appropriate linkers. It revealed that both the vaccines are non-toxic and immunogenic. The tertiary structures of the vaccine proteins were generated, refined, and validated to ensure their suitability. The binding affinity between the vaccine constructs and TLR3 and TLR5 receptors were assessed by molecular docking studies. Molecular dynamics simulations indicated that vaccine construct V1 exhibited greater stability with both TLR3 and TLR5 based on RMSD analysis. Hydrogen bond analysis revealed a stronger binding affinity between the vaccine constructs and TLR5 compared to TLR3. Furthermore, MM-PBSA analysis suggested that both vaccine constructs exhibited a better affinity for TLR5. Considering all aspects, the results suggest that in silico development of CyHV vaccines incorporating multiple epitopes holds promise for management of diseases caused by CyHV-1 and CyHV-3. However, further in vivo trials are highly recommended to validate the efficacies of these vaccines.

## Introduction

Aquaculture is a globally significant and rapidly expanding sector in food production, exhibiting an average annual growth rate of 5.8% from 2001 to 2016^1^. Among the various aquatic species cultivated for human consumption, common carp (*Cyprinus carpio*) stands out as a prominent finfish, ranking as the third most-produced worldwide and accounting for approximately 8% of total global production. As of 2016, the annual production of common carp had reached 4.6 million tons^2,3^. Global fisheries production has increased from 99 to 178 million tonnes during the last 3 decades. Aquaculture’s contribution to global fishing productivity increased from 13% in 1990 to 49% by 2020^4,5^. Conversely, koi carp primarily serves as an ornamental fish, commonly found in personal ponds maintained by hobbyists, and often featured in koi competitions and exhibitions. This species is considered to have cultural importance and is thought to expand enormously depending on the water environment^6,7^. In the late 1990s, both the common carp and koi carp sectors faced a grave threat in the form of a virulent disease caused by *C. herpesvirus* (CyHV), a member of the genus Cyprinivirus, family Alloherpesviridae, and order Herpesvirales^8,9^. Several strains of this virus proved harmful to aquatic animals, with *C. herpesvirus* 3 (CyHV-3), initially known as koi herpesvirus (KHV) or carp interstitial nephritis and gill necrosis virus (CNGV), being the most lethal. Apart from high mortality rates, the ability of KHV to persist throughout the lifetime of infected hosts and its broad host range, which allows for asymptomatic infections, pose serious challenges to carp production and koi breeding worldwide^10^. Similar to other herpesviruses, KHV establishes lifelong infections in its host, potentially reactivating and shedding infectious virus upon exposure to stressors like netting during fish handling operations^11^. It is noteworthy that the World Organisation for Animal Health (WOAH) classifies CyHV-3 as a notifiable disease^12^. The virus was first identified in koi and common carp in Europe and Israel during 1996–1997. Infected fish, even after recovery, can act as carriers of the virus. In the case of KHV-infected koi fish, mortality within the initial 24–48 h of exposure is common^13,14^. Currently, the virus is found in 33 countries, with significant impacts observed on common carp production, a crucial food fish in Israel, Europe, and Asia. The disease generally exhibits a seasonal pattern, occurring in spring or autumn when water temperatures range between 18 and 28 °C^10,15^. Koi fish face a high risk of fatality from the virus, with mortality rates reaching 100%. Symptoms manifest rapidly, usually within 7 to 10 days of exposure to infected fish harboring the virus^16^. Infected koi fish may exhibit excessive mucus secretion, leading to cloudy water in their tanks, along with lethargy, skin lesions, and damage to the gills, spleen, and kidneys. Although microscopic gill lesions are common, intranuclear inclusions are less prevalent compared to other related viruses^13,17^. *Cyprinid herpesvirus* 1 (CyHV-1), causes recurring skin conditions in carp, commonly referred to as “carp pox”. This condition results in the growth of papilloma-like formations, particularly on the fins, but is not fatal. However, it can affect the appearance of show fish^18^. The widely recognized viral pathogen CyHV-1, which causes benign epidermal proliferations in common carp, has also been linked to cutaneous squamous cell carcinoma in koi carp as of recently. CyHV-1 was also identified as the potential causal agent of carp-pox-like disease^19^. In young fish, systemic infections with high mortality rates can occur, and survivors may carry the virus throughout their lives, posing a significant loss if these fish are intended for ornamental purposes^20,21^. Consequently, both *C. herpesvirus* 3 and *C. herpesvirus* 1 have profound ecological and economic impacts on the aquaculture industry. Although these viruses are currently localized to specific regions, their potential for becoming pandemic cannot be discounted^22^. Presently, no effective treatment exists for either strain of the virus, necessitating a focus on preventive measures such as vaccination to control its spread^10^.

In the era of post-genomics, the utilization of computational methods for vaccine design has gained popularity due to the availability of reliable immuno-informatics tools. Epitope-based vaccines offer several advantages over traditional vaccination methods, including high specificity, superior safety, ease of manufacture and storage, and long-lasting efficacy^23^. In silico approaches play a crucial role in identifying the appropriate antigenic epitopes from target proteins for constructing multi-epitope vaccines. This approach enables the discovery of epitopes capable of providing immunity against multiple strains simultaneously, reducing overall costs for farmers by employing a single vaccine to combat both strains. Utilizing various bioinformatics tools, it is possible to predict the highest antigenic scores of epitopes, assess their toxicity and allergenicity, and evaluate their topology. Molecular modeling, analysis of physicochemical properties, docking studies with specific Toll-like receptors (TLRs), and codon adaptation can further validate the findings of in silico research. Molecular dynamics simulations offer insights into the stability, compactness, and affinity of the vaccine with the relevant receptors, providing a better understanding of their interaction. Reverse vaccinology, an advanced method that integrates immunogenomics, immunogenetics, and bioinformatics could serves as a promising approach to develop effective vaccine candidates and mitigate the negative impact of cyprinid viruses on the common carp and koi carp industries. Immunoinformatics approaches have revolutionized vaccine design. By employing reverse vaccinology techniques, it not only significantly cuts down on the time and expense of the development process but also improves the safety and efficacy of the vaccine^24–27^.

The goal of this research was to develop unique multi-epitope subunit vaccines against fish diseases caused by *C. herpesvirus *1 and 3 using computer-assisted methodologies. The developed vaccines will contribute to mitigate the devastating effect caused by *C. herpesviruses*. This study will pave the way to assist the whole aquaculture sector to employ epitope based vaccinations against illnesses of cultures fishes.

## Methods

**Figure 1:**
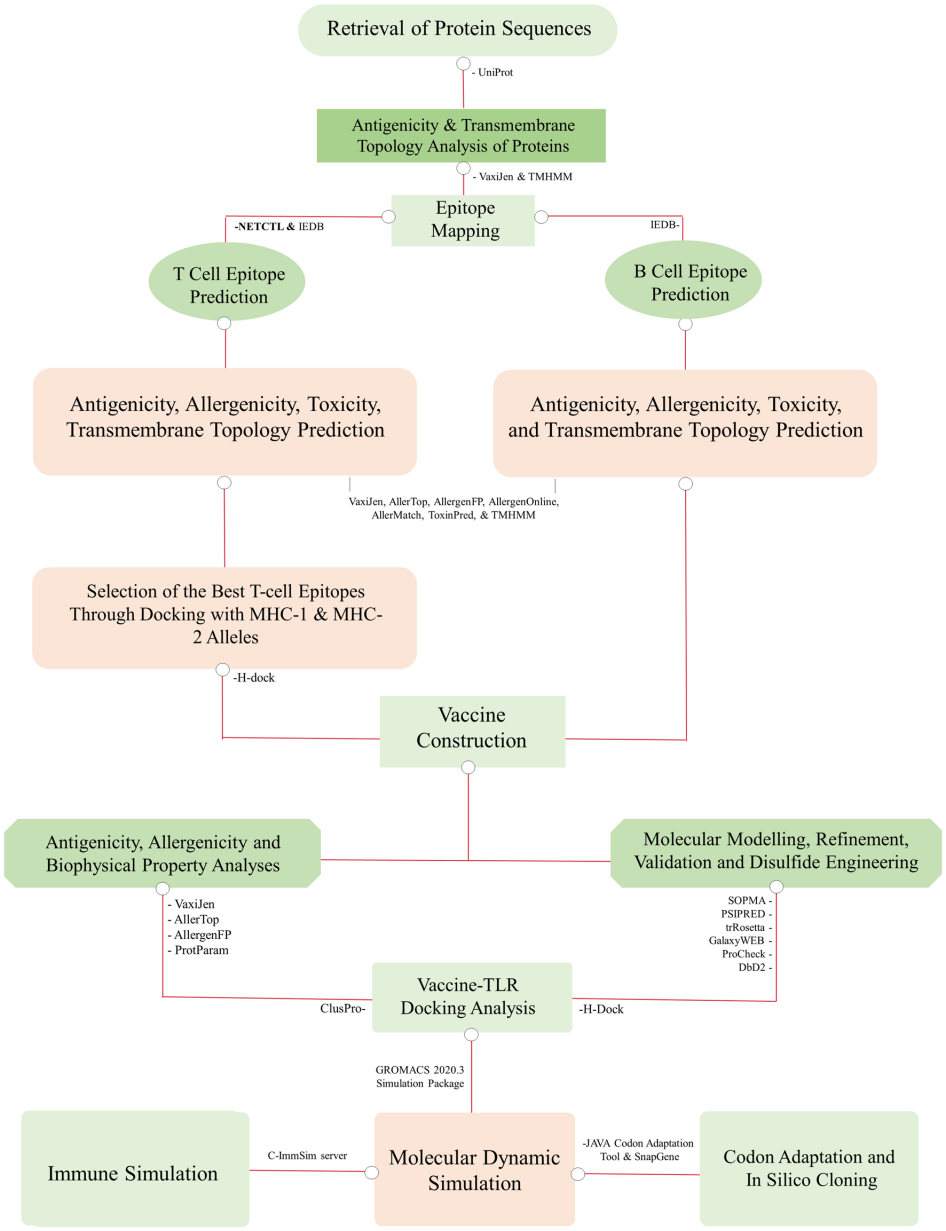
Flowchart of the methodology of the entire study.

The methodology employed in this study is presented in Fig. 1.

### Strain selection and retrieval of protein sequences

Two strains of CyHV-1 and CyHV-3 were selected for this study based on the NCBI database. Comprehensive analysis of both strains was conducted, and the protein sequences of interest were extracted from the UniProt database (https://www.uniprot.org) in FASTA format.

### Antigenicity and transmembrane topology analysis of target proteins

The selected protein sequences from the CyHV-1 and CyHV-3 strains were analysed for antigenicity using the VaxiJen v2.0 online tool (http://www.ddg-pharmfac.net/vaxijen/VaxiJen/VaxiJen.html), with a prediction accuracy threshold of 0.4^28^. To determine the transmembrane topology of the proteins, the TMHMM-2.0 online server (https://services.healthtech.dtu.dk/service.php?TMHMM-2.0) was utilized, keeping all default values^29^.

### Prediction of T-cell epitopes

The NetCTL 1.2 server with Artificial Neural Network (ANN) algorithms was used to predict the epitopes of the specific proteins^30^. The epitopes were analysed for binding with MHC Class-1 super-types, including A1, A2, A3, A24, A26, B7, B8, B27, B39, B44, B58, and B62. The threshold for cumulative score was set at ≥ 1.2 based on proteasomal C-terminal cleavage and Transporter associated with antigen processing (TAP). The epitopes were further evaluated for antigenicity^31^.

### Antigenicity, allergenecity, toxicity, and transmembrane topology analyses

The selected epitopes were assessed for their suitability in vaccine development by predicting their antigenicity, allergenicity, toxicity, and transmembrane topology. Antigenicity was determined using the VaxiJen v2.0 server with a prediction accuracy threshold of 0.4. The TMHMM v2.0 server was employed to predict the topology of the epitopes. Allergenicity analysis was performed using four online servers: AllerTOP v2.0 (https://www.ddg-pharmfac.net/AllerTOP/), AllergenOnline (http://www.allergenonline.org/databasefasta.shtml), Allermatch (https://www.allermatch.org/allermatchsearch/form), and AllergenFP v1.0 (http://ddg-pharmfac.net/AllergenFP/). The toxicity of the epitopes was predicted using the ToxinPred server (http://crdd.osdd.net/raghava/toxinpred/) with default settings and the support vector machine (SVM) approach^32^. The default parameters, threshold value and selection criteria of each of this tool were mentioned in Supplementary Table S1.

### Prediction of 3D structures for enhanced epitopes and molecular docking analysis

The PEP-FOLD server (https://mobyle.rpbs.univ-paris-diderot.fr/cgi-bin/portal.py#forms::PEP-FOLD3) was employed to predict peptide structures for the top-ranked epitopes. These epitopes were chosen based on the structures available in the Protein Data Bank (PDB) database for docking analysis with MHC class I and class II binding epitopes, namely HLA-A*1101 and HLA-DRB-1*0401, respectively. Molecular docking was conducted using the HDock server (http://hdock.phys.hust.edu.cn/), and the resulting docking interactions were visualized using the PyMOL molecular graphics system (https://www.pymol.org/)^33^.

### Identification of B-cell epitopes

Various tools from IEDB were utilized to identify the antigenicity of B cells, employing algorithms such as the Kolaskar and Tongaonkar antigenicity scale^34^, Emini surface accessibility prediction^35^, Karplus and Schulz flexibility prediction^36^, Bepipred linear epitope prediction analysis^37^, Chou and Fasman beta turn prediction^38^, and Parker hydrophilicity prediction^39^. A list of epitope is obtained after submitting the proteins utilizing all those algorithms mentioned, the best b cell epitopes were screened by checking the antigenicity, allergenecity and toxicity.

### Construction of the vaccine, biophysical property and population coverage analysis

To design a multi-epitope vaccine, the best T-cell and B-cell epitopes were combined using various linkers, adjuvants, and the PADRE sequence. In this study, the adjuvant sequence chosen was human beta-defensin-3. Suitable peptide linkers, such as EAAAK, AAY, and GPGPG, were employed to attach the epitopes^40–42^. PADRE sequence is used in the construction to enhance the immune response of CTL epitopes^43^. The antigenicity of the constructed vaccines was predicted using the VaxiJen v.2.0 server. AllerTOP v2.0, AllergenOnline, Allermatch, and AllergenFP v1.0 servers were used to assess allergenecity. The solubility of the vaccine constructs during expression in *Escherichia coli* was evaluated using the Protein-Sol server (https://protein-sol.manchester.ac.uk/)^44^. Additionally, the ProtParam server (http://expasy.org/cgi-bin/protpraram) was employed to assess various physicochemical properties, including aliphatic index, isoelectric pH, molecular weight, GRAVY values, instability index, and estimated half-life of the vaccine construct^45^. For an ideal vaccine candidate, it must be highly antigenic (> 0.4), non-allergenic and having a topology of outside. To determine population coverage of the epitope used the vaccine, we chose East Asia, South Asia, North Asia, Europe and North America utilizing the IEDB population coverage tool (http://tools.immuneepitope.org/tools/population/iedb_input). This website is designed to calculate epitope population coverage in various regions based on the distribution of different MHC alleles to which epitopes bind. The computation was set to class I^46^.

### Secondary and tertiary structure prediction of the vaccine

The secondary structure of the vaccine protein, including alpha-helices, beta sheets, and coil structures, was predicted using the PSIPRED v3.3 server (http://bioinf.cs.ucl.ac.uk/psipred/) and SOPMA (https://npsa-prabi.ibcp.fr/cgi-bin/npsa_automat.pl?page=/NPSA/npsa_sopma.html)^47,48^. To predict the tertiary structure of the constructed vaccine, the trRosetta server (https://yanglab.nankai.edu.cn/trRosetta/) was utilized^49^. Subsequently, the GalaxyWEB server (http://galaxy.seoklab.org/) was employed to refine these models^50^. The refined models were further validated using the Saves v6.0 server (https://saves.mbi.ucla.edu/) by examining the ERRAT value and Ramachandran plot of each model^51^. The refined vaccine was also evaluated using the ProSA server (https://prosa.services.came.sbg.ac.at/prosa.php) to analyse the z-score graph^52^.

### Disulphide engineering and molecular docking analysis of the vaccine

Disulphide linkages were introduced into the anticipated vaccine structures using the DbD2 server (http://cptweb.cpt.wayne.edu/DbD2/). Disulphide engineering improves protein stability. Cysteine residues were strategically placed in the highly mobile regions of the protein, allowing for the formation of disulfide bonds in the streamlined structure. During the analysis, the Cα–Cβ–Sγ angle remained at 114.6° ± 10, whereas the χ^3^ angle was adjusted at − 87° or + 97°. Chi3 is formed when Cβ–Sγ–Sγ–Cβ bonds rotate around the Sγ–Sγ bond. Surface-exposed residues were associated with increased B-factor levels. There is a strong correlation between residue depth and the B-factor. Individual amino acids were converted into cysteine, and suitable pairs of residues capable of forming disulfide bonds were identified^53,54^. The constructed vaccines V1 and V2 were evaluated for their binding affinity against TLR3 (PDB ID: 2A0Z) and TLR5 (PDB ID: 3J0A) receptors. Molecular docking was performed using the Server HDock and ClusPro (https://cluspro.bu.edu/login.php)^55^. Subsequently, the 3D structures of the complexes were visualized and analyzed using the PyMOL v2.0 software^56^.

### Molecular dynamics simulation studies and MM-PBSA calculation

In the 100 ns molecular dynamics (MD) simulation studies, the docking of two vaccine constructs, V1 and V2, with TLR3 and TLR5 resulted in the formation of four complexes: TLR3-V1 complex, TLR3-V2 complex, TLR5-V1 complex, and TLR5-V2 complex. The MD simulations were performed using the Gromacs-2020.4^57,58^ program on the HPC cluster at Bioinformatics Resources and Applications Facility (BRAF), C-DAC, Pune. The vaccine constructs V1, and V2 have 169 and 167 residues, respectively. TLR3 has one chain (chain A), while TLR5 has two chains (chains A and B). The input topologies of vaccine constructs and TLRs were constructed using the CHARMM-36 force field parameters^59,60^. The TIP3P water model^61^ was used to solvate the complexes by placing them in a box of dodecahedron unit cells, keeping the system's edges 1 nm away from the box. The systems were neutralized with the addition of appropriate counter-ions, where TLR3-V1 needed 11 chloride ions, TLR3-V2 system needed 16 chloride ions, TLR5-V1 system needed one sodium ion, and TLR5-V2 systems needed 4 chloride ions. The resultant systems were energy minimized with the steepest descent algorithm until the force-constant reached the 100 kJ mol-1 nm-1 threshold. The systems were later subjected to equilibration initially at constant volume and constant temperature (NVT) conditions of 300 K which was achieved using a modified Berendsen thermostat^62^, and later at constant volume and constant pressure (NPT) conditions of 1 atm pressure which was achieved using Berendsen barostat^63^. The NVT and NPT equilibrations were carried out for 1 ns each. The equilibrated systems were subjected to the 100 ns production phase MD simulations. During the production phase MD simulation, the temperature conditions of 300 K were achieved with a modified Berendensen thermostat, pressure conditions of 1 atm were achieved with the Parrinello-Rahman barostat^64^, and the covalent bonds were restrained with the LINCS algorithm^65^. During simulations, the long-range electrostatic energies were computed with Particle Mesh Ewald (PME) method^66^ with a cut-off of 1.2 nm. Post simulations, the periodic boundary conditions (PBC) were removed from the output trajectories, and such trajectories were used in the analysis.

The MD analysis includes the analysis of the root mean square deviations (RMSD) in the C-α atom of TLR chains and vaccines. The root mean square fluctuation (RMSF) in the side chain atom of each TLR chain and vaccine residue was also analyzed. The analysis of the radius of gyration (Rg) on each chain of TLR and bound vaccine chain was performed separately to analyze the system's compactness and stability. The interchain hydrogen bond formation was analyzed. The hydrogen bonds between TLR3 chain A and the vaccine were analyzed here. In the case of TLR5 vaccine complexes, as the vaccine is bound at the interface of two chins, the hydrogen bonds between TLR5 chain A and vaccine and TLR5 chain B and vaccine were separately analyzed. These interchain hydrogen bonds were further visually inspected in the trajectories isolated at equilibrium state, 25, 50, 75 and 100 ns simulation period in the ChimeraX program^67^. The mean smallest distance between residue pairs in each complex was analyzed using the gmx_mdmat program. The mean smallest distances between residues pairs thus obtained were used to construct the contact maps^68^. The major path of motions in each complex was analyzed from principal component analysis (PCA)^69^. In PCA, the gmx covar program was used to obtain the covariance matrix for the C-α atom of each complex and after diagonalizing this covariance matrix using the gmx anaeig program, the eigenvectors and eigenvalues were obtained. Here the eigenvectors represent the motion path, while eigenvalues represent the mean square fluctuation.

Further, the first two principal components (PC1 and PC2) obtained were used in Gibb’s free energy landscape (Gibb’s FEL) analysis^69^ using the gmx sham program. The change in the secondary structures was analyzed from the definition of the protein secondary structure (DSSP) program^70,71^. Further, the correlation between the extent of fluctuations and displacements of side chains residues of TLR chain and vaccine was analyzed from a dynamical cross-correlation matrix (DCCM)^72^. Molecular mechanics energies combined with Poisson Boltzmann surface area continuum solvation (MM-PBSA)^56,73^ calculations were carried out on each TLR-vaccine complex. Different interaction energies and the binding free energies between TLR chains and vaccines were estimated in MM-PBSA calculations. The trajectories isolated at every 1 ns were used in MM-PBSA calculations. The images of protein structures were rendered in ChimeraX^67^, PyMOL^56^, and VMD^73^, and graphs were plotted in XMGRACE^74^. Gibb’s FEL plots were generated using the Python-based Matplotlib package^75^. The DCCM analysis was performed in R statistical program^76^ using the Bio3D package^77^.

### Codon adaptation and in-silico cloning study

The codon adaptation index and the coding sequence of the predicted vaccine were determined using the J-CAT server (http://www.jcat.de) and the *E. coli* strain K12 was selected as the host. Furthermore, the SnapGene tool (from GLS Biotech, available at snapgene.com) was utilized for cloning and generating vaccines, providing valuable insights into epitope-based peptide vaccines. When utilizing the server, the prokaryotic ribosome binding site, BglII and Apa1 cleavage sites, and Rho independent transcription termination were avoided. The most optimal vaccine construct sequence was then inverted, and the N- and C-terminal BglII and Apa1 restriction sites were conjugated using the SnapGene software. It was also employed to design a recombinant plasmid for further experimentation and analysis^78^. The pET28a(+) was used as the expression vector because it has been used in numerous investigations because it can be expressed in the *Escherichia coli* system. The pET28a(+) plasmid also contained a kanamycin resistance gene and a LacI gene to aid in the selection and differentiation of the *E. coli*^79^*.*

### Immune simulation studies

The immune simulation of the vaccine was performed utilising the C-ImmSim web server (http://150.146.2.1/C-IMMSIM/index.php), which provides a realistic immune interaction prediction^80^. The immune simulation kept all parameters default except for time steps (set at 1, 84, and 170), and the number of simulation steps was set at 1,050. The vaccine's suggested dosage is three injections every 4 weeks, which corresponds to the proper period between doses for all commercial vaccines^81,82^.

## Results

### Selection and retrieval of target protein

For each strain of CyHV-1 and CyHV-3, three highly antigenic outer-membrane proteins were carefully chosen. The target proteins selected from CyHV-1 included Membrane protein ORF25 (Accession ID: K7PBK6), Protein ORF136B (Accession ID: K7PCA0), and Major capsid protein (Accession ID: Q52UN7). From CyHV-3, Glycoprotein (Accession ID: K9JF86), Capsid triplex subunit 1 (Accession ID: A3QMN4), and Protein ORF104 (Accession ID: A3QMS2) were selected. The transmembrane topology of these proteins was evaluated using the TMHMM server, while their antigenicity was assessed using VaxiJen server 2.0. Supplementary Table S2 provides comprehensive details of the selected proteins, including their accession IDs, antigenicity, and transmembrane topology.

### T-cell epitope prediction

After analysing the proteomic data of the selected proteins, NetCTL 1.2 server was utilized to predict T-cell epitopes. For the selected proteins of CyHV-1, Membrane protein ORF25 yielded 687 epitopes, Protein ORF136B had 217 epitopes, and Major capsid protein had 1261 epitopes. In the case of CyHV-3, Glycoprotein yielded 508 epitopes, Capsid triplex subunit 1 had 388 epitopes, and Protein ORF104 produced 650 epitopes. Subsequently, the predicted epitopes of each protein were subjected to VaxiJen v2.0 server to predict their antigenicity, and TMHMM was employed to determine their topology. Considering parameters such as toxicity, allergenecity, antigenicity, and transmembrane topology, the four best epitopes from each protein were chosen for docking with MHC alleles. Supplementary Table S3 provides a comprehensive list of the selected epitopes of CyHV-1 and CyHV-3, along with their respective allergenecity, antigenicity, topology, and toxicity.

### Docking analysis with MHC alleles and selection of T-cell epitopes

To analyse the interactions between the previously selected top 24 epitopes (12 from CyHV-1 proteins and 12 from CyHV-3 proteins) and two HLA alleles, namely HLA-A*1101 and HLA-DRB-1*0401, 3D structures were predicted. The PEP-FOLD3 server generated five 3D structures for each epitope, and the most optimal one was identified for the subsequent docking study. Upon performing docking using the H-dock server, the majority of putative T-cell epitopes from both proteins exhibited a strong binding affinity against both HLA-A*1101 and HLA-DRB-1*0401 (Supplementary Table S4). The epitope FKPLEWCSG displayed the highest binding score of -214.50 against HLA-A*1101, while the epitope LTFKPNWQP demonstrated the highest binding score of -217.80 against HLA-DRB-1*0401. Selection of T-cell epitopes for vaccine construction was based on their binding affinity. Table 1 provides a comprehensive list of the top epitopes, including their docking scores and root mean square deviation values and Supplementary Fig. S1 provides a visual representation of the docking.

**Table 1:** Docking result of best T cell epitopes against HLA-A*1101 and HLA-DRB-1*0401.

MHC Allele	Epitope	Docking score (Kcal/mol)	RMSD (nm)
HLA-A*1101	FKPLEWCSG	− 214.50	4.955
	LTFKPNWQP	− 198.30	5.125
	TWENMEFSY	− 190.98	4.886
	CPFKPLEWC	− 184.20	4.852
	LEPAWVDPR	− 178.31	1.192
	PFKPLEWCS	− 178.24	2.326
HLA-DRB-1*0401	LTFKPNWQP	− 217.80	4.630
	TWENMEFSY	− 212.82	5.003
	PFKPLEWCS	− 209.54	4.595
	CPFKPLEWC	− 207.45	4.687
	FKPLEWCSG	− 201.98	5.045
	LEPAWVDPR	− 183.90	4.990

### B cell epitope selection

The prediction of B cell epitopes involved the utilization of IEDB's six algorithms. Based on their antigenicity, allergenecity pattern, and toxicity analysis, the top two B cell epitopes for each protein of both CyHV-1 and CyHV-3 were ultimately selected for vaccine design. The selected B cell epitopes, along with their corresponding antigenicity, transmembrane topology allergenicity, and toxicity, are listed in Supplementary Table S5.

### Construction of the vaccine and biophysical property analysis

The vaccine construction focused on epitopes that exhibited high antigenicity, non-allergenicity, non-toxicity, and conservation across the population. Two vaccine molecules, designated as V1 and V2, were constructed. The arrangement of T cell and B cell epitopes, along with the N-terminal beta-defensin-3 adjuvant and the PADRE sequence, was achieved using various linkers such as EAAAK, AAY, and GPGPG. Each construct comprised four T cell epitopes and five B cell epitopes. The designed vaccine constructs, V1 and V2, had lengths of 169 and 167 residues, respectively. The schematic and constructive diagrams of the vaccine constructs can be seen in Supplementary Fig. S2.

### Evaluation of physicochemical properties and population coverage

The ProtParam tool was employed to assess the physicochemical properties of the constructed vaccines. V1 demonstrated a theoretical isoelectric point (pI) of 9.79, an aliphatic index of 48.70, and a GRAVY value of − 0.802. On the other hand, V2 displayed a theoretical pI of 10.07, an aliphatic index of 63.29, and a GRAVY value of − 0.506. Both vaccines were determined to be stable, with an average half-life of 30 h. Furthermore, their solubility exceeded 0.45, indicating good solubility. In addition, both vaccines were predicted to be probable antigens, with V1 exhibiting an antigenicity score of 0.9457 and V2 scoring 0.8430. Both vaccines were also predicted to be non-allergenic. The biophysical properties of the vaccine constructs are presented in Supplementary Table S6. The MHC1 epitopes of the vaccine construct were predicted and MHC class 1 supertypes alleles were used to uncover the population coverage. Where an average of 83% coverage was found for the selected epitopes. The overall result were elucidated in Supplementary Table S7 and Supplementary Fig. S3.

### Secondary and tertiary structure prediction of the vaccine

The secondary structure prediction was conducted using the SOPMA Server. The results revealed that V1 consisted of 45 residues (26.63%) in an alpha-helix state, 27 residues (15.98%) in a beta-sheet state, 10 residues (5.92%) in a turn state, and 87 residues (51.48%) in a coil state. Similarly, V2 comprised 50 residues (29.94%) in an alpha-helix state, 23 residues (13.77%) in a beta-sheet state, 12 residues (7.19%) in a turn state, and 82 residues (49.10%) in a coil state. Supplementary Figs. S4 and S5 display the secondary structure predictions of both vaccines as determined by the Psipred server.

The 3D structure of the constructed vaccines was generated using the trRosetta server. Subsequently, the best-predicted vaccine models were further refined using the GalaxyWeb server. The SAVESv6.0 server was employed to select the optimal model. Vaccine V1 exhibited an ERRAT value of 76.875 and a favored region of 74.8% in the Ramachandran plot, while Vaccine V2 displayed an ERRAT score of 75.4, with the favored region comprising 65.9%. The Z scores for V1 and V2 were determined to be − 5.18 and − 5.07, respectively. The 3D models, ERRAT maps, Ramachandran plots, and z score maps of V1 and V2 can be observed in Supplementary Figs. S6 and S7.

### Disulphide engineering of the vaccine

In the disulphide engineering process, 27 pairs of amino acid residues were identified in V1, while V2 had 20 pairs capable of forming disulphide bonds. After evaluating the chi3 and B-factor parameters of the residue pairs based on energy, two pairs (TRP 74-PRO 161 and ASN 86-GLY 149) for V1 and one pair (ARG 121-GLY 134) for V2 were selected. These pairs were modified with cysteine. The chi3 values for residue screening ranged from − 87 to + 97, and the energy values were less than 2.5. Supplementary Figs. S8 and S9 provide a visualization of the selected disulphide bond pairs.

### Molecular Docking Analysis of the Vaccine

**Figure 2:**
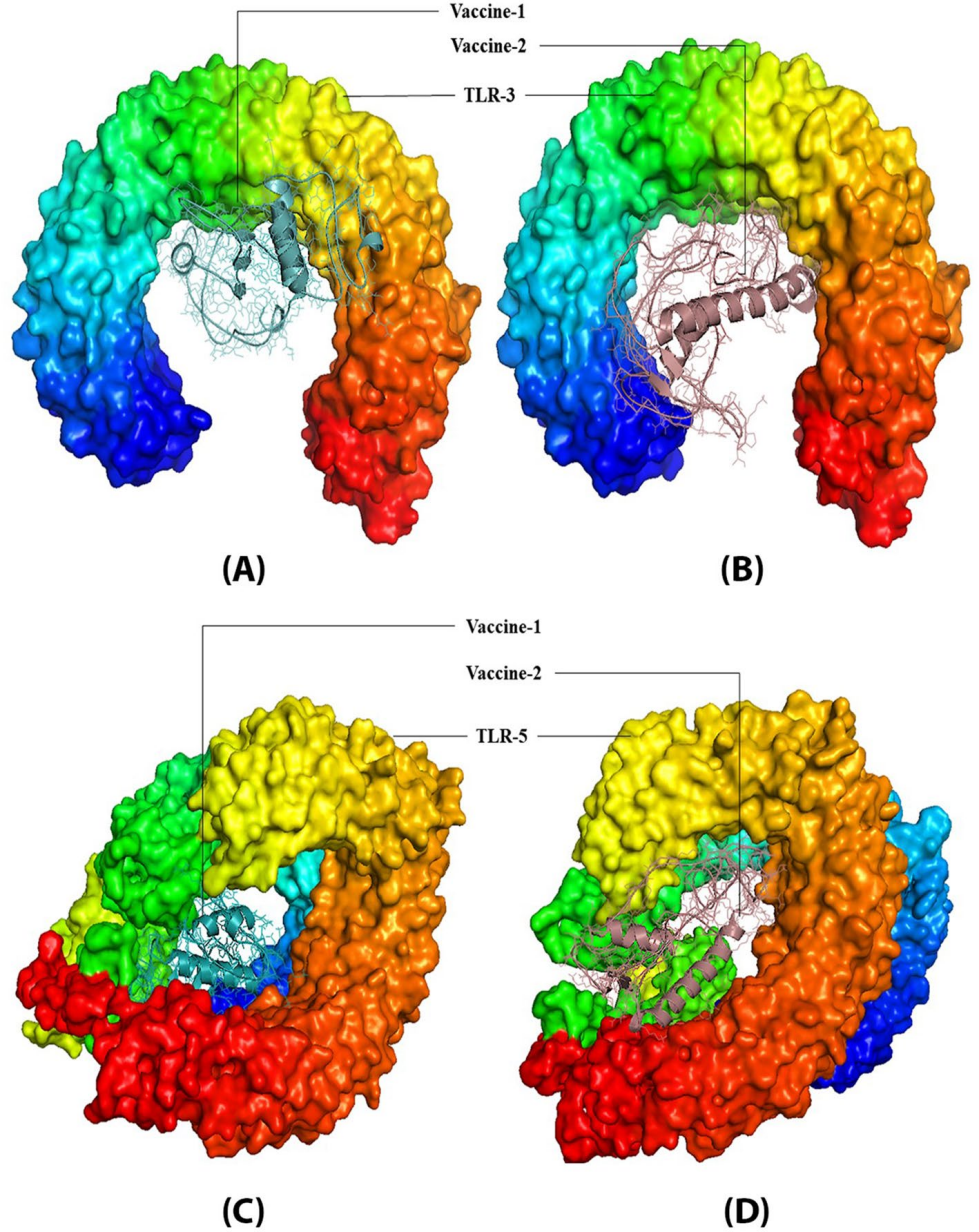
3D model of TLR3 Receptor with (**A**) Vaccine V1 (**B**) Vaccine V2**,** 3D model of TLR5 Receptor with (**C**) Vaccine V1 (**D**) Vaccine V2.

Following the disulphide engineering, both vaccines were subjected to molecular docking with TLR3 and TLR5. The docking analysis was performed using the Hdock and ClusPro servers. Both V1 and V2 exhibited high binding affinity in both studies, as indicated in Table 2. V2 demonstrated the highest affinity with TLR5, displaying the lowest energy of − 1331.8. V1 also exhibited a low energy value of -1226.9. Three-dimensional models of the constructed vaccines with TLR3 and TLR5, generated by Pymol, are depicted in Fig. 2.

**Table 2:** Docking score of vaccine construct V1 and vaccine construct V2 with TLRs.

Protein	Vaccine	H-dock Docking Score (Kcal/mol)	Cluspro Docking energy (Kcal/mol)
TLR3 (PDB ID: 2A0Z)	v-1	− 289.09	− 731.0
	v-2	− 278.26	− 997.2
TLR5 (PDB ID: 3J0A)	v-1	− 319.28	− 1226.9
	v-2	− 339.60	− 1331.8

### Molecular dynamics studies and MM-PBSA calculation

#### Root Mean square deviation evaluation

**Figure 3:**
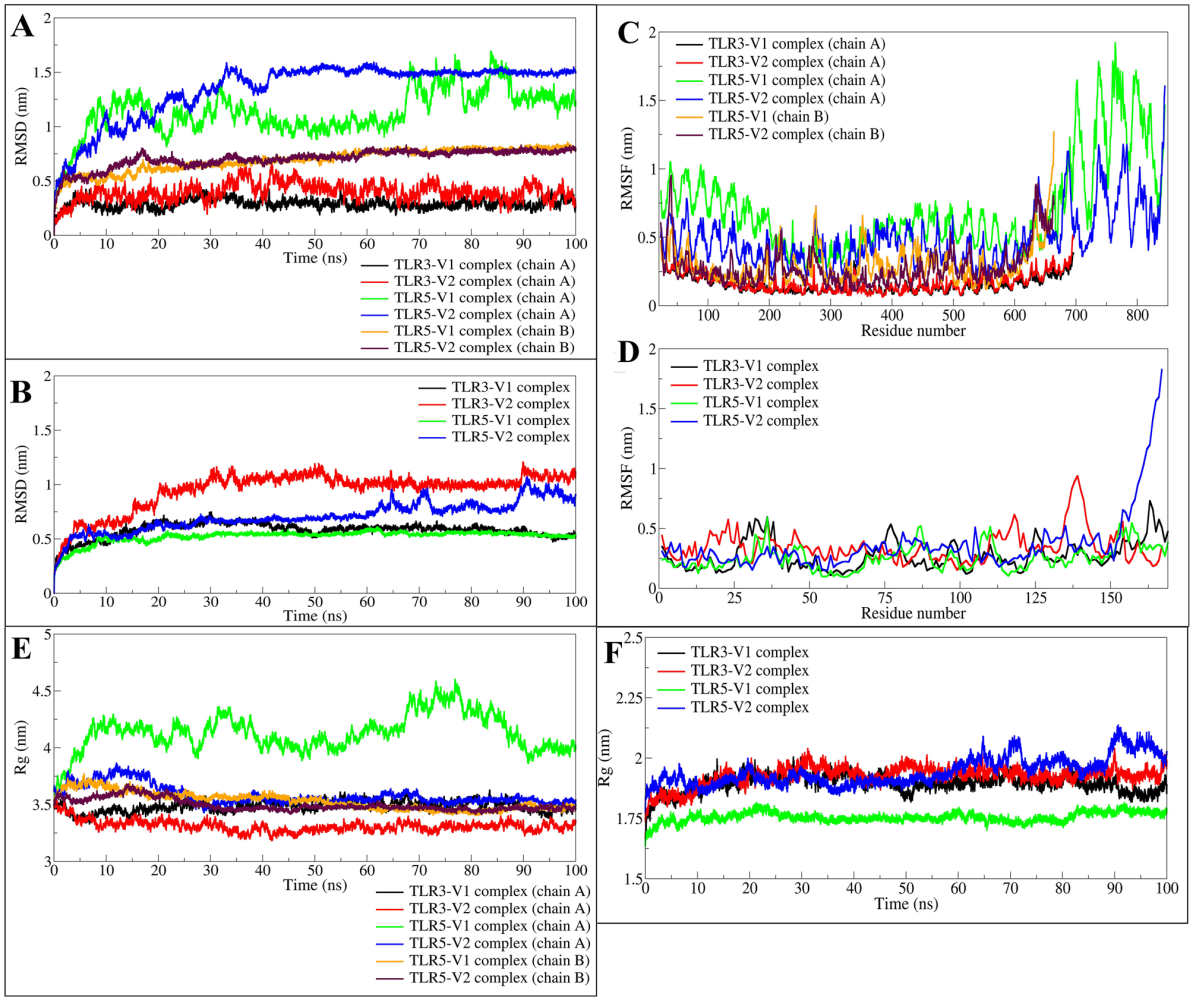
The RMSD analysis. (**A**) RMSD plot for chains of TLR3 and TLR5, and (**B**) RMSD plot for vaccine bound to respective TLRs as shown in the figure legends. The RMSF inside chain atoms of residues. (**C**) RMSF in TLR chains, and (**D**) RMSF in vaccines bound to TLR3 or TLR5. The radius of gyration analysis. (**E**) Rg in TLR chains, and (**F**) Rg in vaccines bound to respective TLRs.

In the case of TLR3 vaccine complexes, the TLR3 chain A bound to vaccine-V1 has considerably fewer fluctuations compared to TLR3 chain A bound to vaccine-V2, where the average RMSDs are 0.2912 and 0.3975 nm, respectively (Fig. 3A, Table 3). The chain A in TLR3 bound to vaccine V1 showed reasonably converged and stable RMSD throughout the simulation. While chain A of TLR3 bound to vaccine V2 showed deviations until the first 50 ns simulation, stabilising with a lower magnitude of deviations thereafter until the end of the simulation. In the case of TLR5 vaccine complexes, chain A in both complexes has a higher magnitude of fluctuations with averages of 1.1359 and 1.3442 nm, respectively. Specifically, chain A of TLR5 bound to vaccine-V1 showed larger fluctuations throughout the simulation period. Chain A of TLR5 bound to vaccine V2 showed a rise in RMSD, reaching a maximum RMSD of around 1.5 nm at around 35 ns and stabilising thereafter to a constant RMSD of around 1.5 nm until the end of the simulation period. Chain B in these complexes has stable RMSD with averages of 0.6934 and 0.7056 nm, respectively. Chain B in both complexes coincidently showed an almost similar magnitude of RMSD after around 40 ns simulation.

**Table 3:** Estimates of averages for different MDS analysis parameters.

Details of the complexes	Average (nm)			Average number of hydrogen bond with respective vaccine
	RMSD in C-α atoms	RMSF	Gyrate	
TLR3-V1 complex				
Chain A	0.2912 (0.0445)	0.1571 (0.0618)	3.4845 (0.0457)	11.64 (3.02)
Vaccine	0.5784 (0.0700)	0.2748 (0.1246)	1.8945 (0.0363)	–
TLR3-V2 complex				
Chain A	0.3975 (0.0811)	0.1699 (0.0708)	3.3122 (0.0491)	8.86 (2.58)
Vaccine	0.9494 (0.1750)	0.3470 (0.1331)	1.9261 (0.0401)	–
TLR5-V1 complex				
Chain A	1.1359 (0.2081)	0.7121 (0.3324)	4.1323 (0.1614)	6.88 (2.40)
Chain B	0.6934 (0.1034)	0.2968 (0.1374)	3.5313 (0.0751)	11.78 (3.77)
Vaccine	0.5228 (0.0565)	0.2550 (0.1034)	1.7546 (0.0203)	–
TLR5-V2 complex				
Chain A	1.3442 (0.2632)	0.4916 (0.2003)	3.5810 (0.0824)	2.91 (1.45)
Chain B	0.7056 (0.0819)	0.2813 (0.1407)	3.4969 (0.0523)	13.75 (2.77)
Vaccine	0.7045 (0.1337)	0.3633 (0.2530)	1.9442 (0.0580)	–

Standard deviations in average values are given in parentheses.

In the case of TLR3 vaccine complexes, the RMSD in vaccine-V1 is considerably lower compared to vaccine-V2, with an average RMSD of 0.5784 and 0.9494 nm, respectively (Fig. 3B). The RMSD in vaccine-V1 bound to TLR3 stabilizes after around 40 ns, while in vaccine-V2, it stabilizes after the initial fluctuations until the first 50 ns simulation period. The RMSD in vaccine-V2 bound to TLR5 is stable until around 60 ns; thereafter, considerable fluctuations until the end of the simulation were observed with an average RMSD of 0.7045 nm. The RMSD in vaccine-V1 bound to TLR5 is the least among other bound vaccines and stable throughout the simulation with an average RMSD of 0.5228 nm.

#### Root mean square fluctuation evaluation

Chain A of TLR3 showed minimal and almost similar RMSF in all the residues with average RMSF of 0.1571 and 0.1699 nm, respectively (Fig. 3C). On the other hand, chain A of TLR5 showed a larger magnitude of fluctuations. Specifically, chain A of TLR5 bound to vaccine-V1 has the highest fluctuations with an average RMSF of 0.7121 nm. Here, the many residues up to residue 625 from the TLR5 bound to vaccine-V1 has an RMSF well below 1 nm, while residues beyond 625 have RMSF reaching beyond 1.5 nm. The chain B of TLR5 bound to the vaccine showed almost similar RMSF in both complexes. The residues in the range of 200–300 in both vaccines bound to TLR5 showed some major fluctuations. The vaccine-V1 bound to TLR5 additionally showed fluctuations in the residues ranging from 325 to 450.

Varied fluctuations were observed in the vaccine residues (Fig. 3D). Typically, it was found that vaccine-V1 has lower fluctuations, with an average of 0.27480.2968 nm, while vaccine-V2 has slightly higher fluctuations, with an average of 0.3470–0.3633 nm. Further, the residues in the 25–35, 75–80, and 120–130 ranges from vaccine-V1 bound to TLR3 showed major fluctuations. While the residues in the ranges 30–40, 80–90, and 110–120 from vaccine-V1 bound to TLR5 showed major fluctuations. Interestingly, the vaccine-V2 bound to TLR3 showed fluctuations in almost all residues and major fluctuations were seen in the residues in the range 110–140. Contrary to this, the vaccine-V2 bound to TLR5 showed a lower magnitude of fluctuations in all the residues.

#### Radius of gyration evaluation

In the TLR5-V1 complex, the TLR5 chain A has the highest total radius of gyration (Rg), reaching a maximum Rg of 4.5 nm at around 75 ns and an average of 4.1323 nm (Fig. 3E). While in the TLR3-V2 complex, the TLR3 chain A has the lowest total Rg with an average of 3.3122 nm. The TLR chains in other complexes, viz*.* TLR3-V1, TLR5-V1, and TLR5-V2 were found stable and almost similar total Rg after around 25 ns simulation period.

The vaccine-V1 bound to TLR5 showed the least total Rg, averaging 1.7546 nm. The vaccine-V2 bound to TLR5 showed deviations in total Rg after around 60 ns with an overall average total Rg of 1.9442 nm (Fig. 3F). The total Rg for vaccine-V1 and vaccine-V2 remained stable throughout the simulation, with a few minor deviations with an average of 1.8945–1.9261 nm.

#### Hydrogen bond analysis

**Figure 4:**
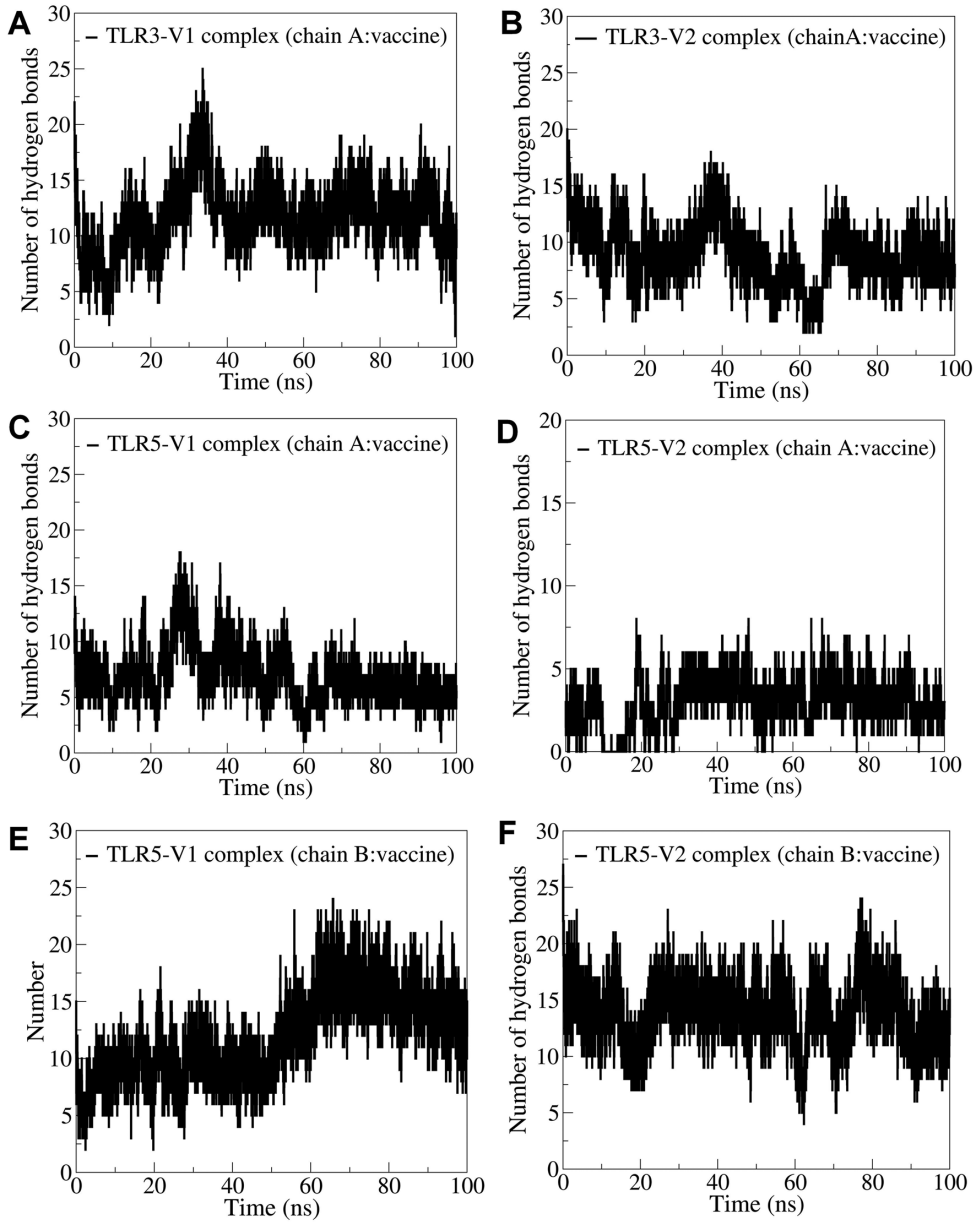
Hydrogen bond analysis. Interchain hydrogen bonds between (**A**) TLR3 and vaccine-V1, (**B**) TLR3 and vaccine-V2, (**C**) TLR5 chain A and vaccine-V1, (**D**) TLR5 chain A and vaccine V2, (**E**) TLR5 chain B and vaccine V1, and (**F**) TLR5 chain B and vaccine V2.

The TLR3 has a single chain, chain A, while TLR5 has two chains, chain A and chain B. In the case of TLR5, the vaccine constructs are bound at the interface of chains A and B. The interchain hydrogen bonds between these chains of TLR and vaccine constructs were analyzed separately. In the TLR3-V1 complex, around 10 hydrogen bonds are consistently formed between TLR3 and vaccine-V1 (Fig. 4A). More hydrogen bonds reaching a maximum of 25 were observed forming at around 25 ns, and thereafter around 10 hydrogen bonds remained stably formed. In the case of vaccine-V2 bound to TLR3, slightly fewer hydrogen bonds were formed with an average of around 8 hydrogen bonds being formed consistently (Fig. 4B). In this complex, the initial 10 ns simulation period and simulation period of 35–45 ns showed more hydrogen bonds than the rest of the simulation period. In the TLR5 vaccine complex, the vaccine being in close contact with chain B forms more hydrogen bonds with this chain. With TLR5 chain A, vaccine-V1 forms around 6 and vaccine-V2 forms around 2 hydrogen bonds (Fig. 4C,D). Here, the vaccine-V1 forms more hydrogen bonds, reaching a maximum of around 17 hydrogen bonds during the 20–40 ns simulation period, while the rest showed around 6 hydrogen bonds. Vaccine-V1 also formed around 11 consistent hydrogen bonds with TLR5 chain B (Fig. 4E). Interestingly, the first 60 ns simulation showed around 10 hydrogen bonds formed. In comparison, the rest of the simulation period showed more than 10 hydrogen bonds, reaching a maximum of around 22 hydrogen bonds formed occasionally during this simulation period (Fig. 4F). In the case of vaccine-V2 bound to TLR5 chain B around 13 hydrogen bonds were formed. However, during a brief period at around 20 ns, 60 ns, and 75 ns, slightly fewer than 13 hydrogen bonds were observed.

**Figure 5:**
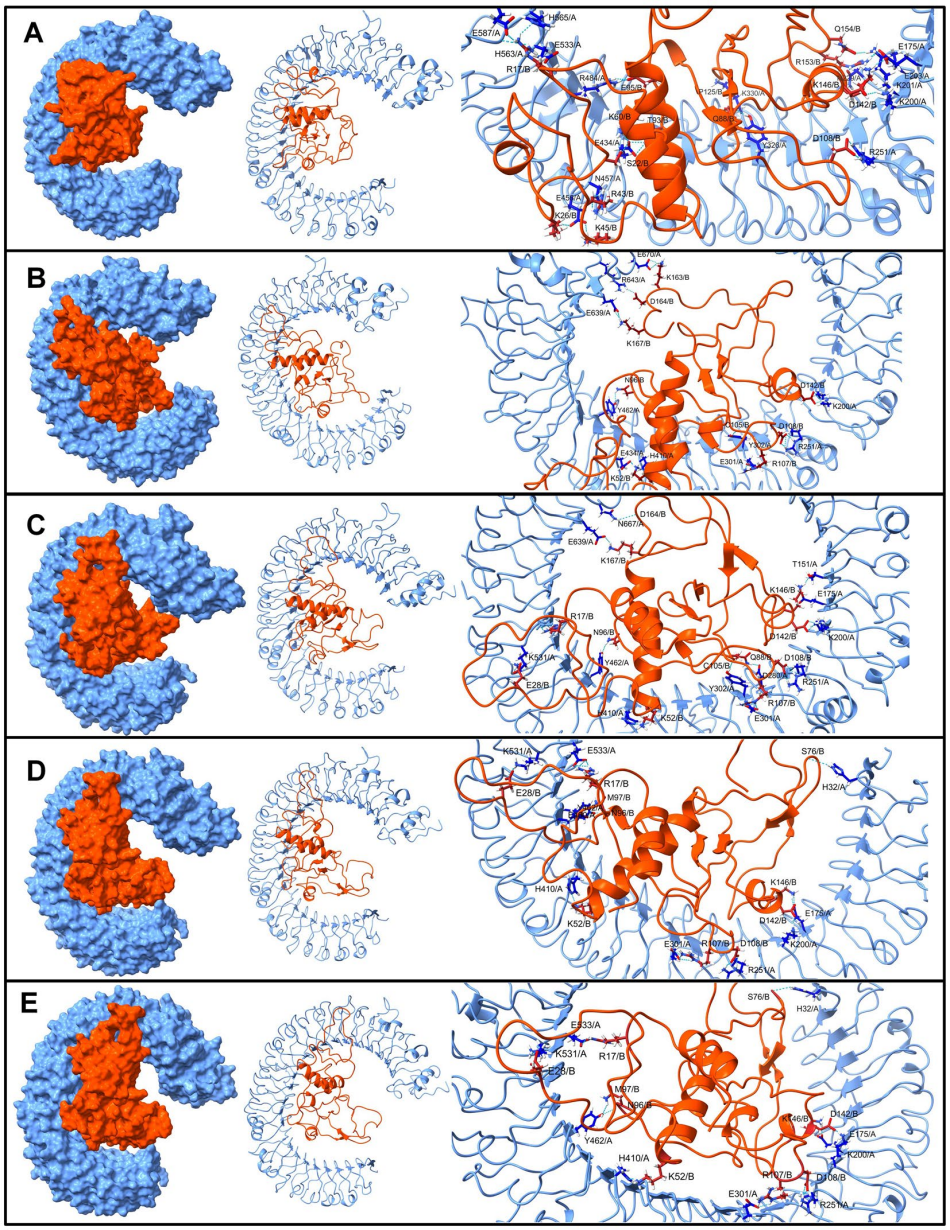
The inter-chain hydrogen bonds between TLR3 and vaccine-V1 (TLR3 surface/cartoon are shown in light blue while the vaccine is in brown color).

The interchain hydrogen bonds were further investigated in the equilibrated trajectory and trajectories isolated at 25, 50, 75, and 100 ns simulation. In the TLR3-V1 complex, the vaccine residues Arg17, Ser22, Lys26, Arg43, Lys45, Lys60, Thr93, Lys146, Arg153, Asp142, Gln154, Asp108, Gln88, Pro125, and Glu95 formed a hydrogen bond with TLR3 residues Lys200, Lys201, Arg251, Tyr326, Lys330, Arg484, and Glu533 (Fig. 5). Most of these hydrogen bonds broke during the first 25 ns simulation period. Except the hydrogen bonds with residues Asp142 and Asp108 of vaccine-V1 and Lys200 and Arg251 of TLR3, a new hydrogen bonds between vaccine-V1 residues Lys52, Asn96, Arg107, Lys163, Lys167, Cys105, and Asp164 and TLR3 residues Tyr302, Arg643, His410, Glu434, Tyr462, Glu301, Glu670, and Glu639 were seen formed during this 25 ns simulation period. Most of these hydrogen bonds remained stable. The hydrogen bonds between TLR3 residues Lys200, Arg251, Tyr302, His410, and Glu301 and vaccine-V1 residues Lys52, Asn96, Arg107, and Asp142 were found stable throughout the rest of the simulation period. During the 50 ns onward simulation period, the hydrogen bonds between TLR3 residues Lys531, Tyr462, and Glu175 and vaccine-V1 residues Arg17 and Glu28 remained stable in addition to the above-mentioned hydrogen bonds.

**Figure 6:**
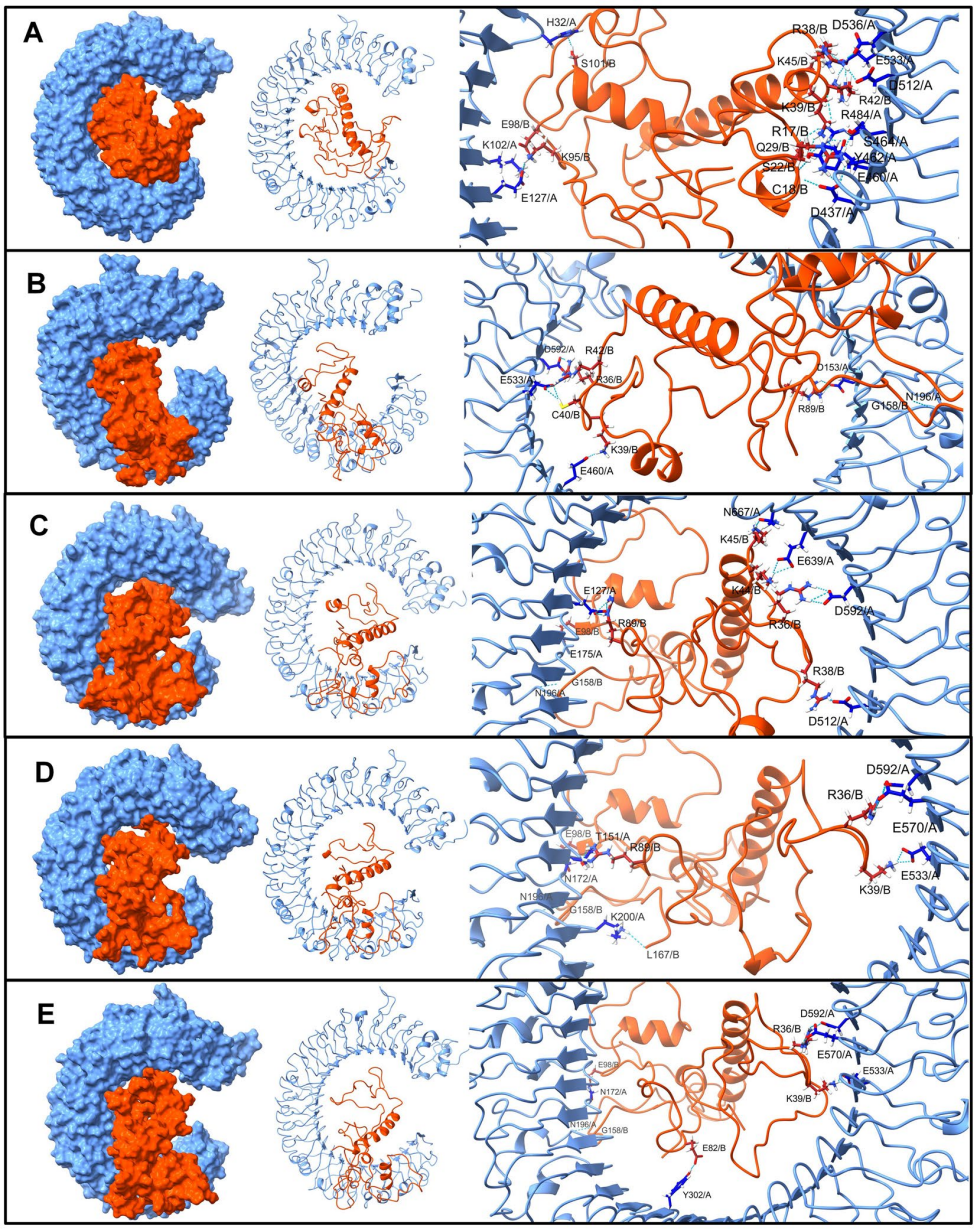
The inter-chain hydrogen bonds between TLR3 and vaccine-V2. (Same color scheme as Fig. 6).

In the case of the TLR3-V2 complex, the equilibrated trajectory showed that the hydrogen bonds formed between the TLR3 residues Lys102, Tyr462, Arg484, Asp437, Ser464, Asp437, Glu460, Asp512, Asp512, Asp536, Glu460, Tyr462, Glu533, Tyr462, Glu533, Glu127, and His32 and vaccine-V1 residues Arg17, Cys18, Ser22, Arg38, Lys39, Arg42, Lys45, Lys95, Ser101, Glu98, Ser22, Gln29, and Arg42 (Fig. 6). Amongst these hydrogen bonds, the hydrogen bonds between TLR3 residue Asn196 and vaccine-V1 residues Arg36, Lys39, and Gly158 remained intact until the end of the simulation. The trajectory isolated at 25 ns showed the new hydrogen bonds between TLR3 residues Asp592 and Asp153 and vaccine-V1 residues Cys40, Arg42, and Arg89. The trajectory isolated at 50 ns showed a new hydrogen bond in addition to these between the TLR3 residues Asn175, glu639, Asn667, Glu127 and vaccine-V1 residues Arg38, Lys44, Lys45, and Glu98. Few new hydrogen bonds were observed in the trajectory isolated at 75 ns, including the new hydrogen bonds between the TLR3 residues Lys200, Glu570, Glu533, Thr151 and vaccine-V1 residues Arg89 and Leu167. The last 100 ns trajectory showed the new hydrogen bonds and some stable ones with the TLR3 residues Tyr302 and Glu533 and vaccine-V1 residues Glu98 and Glu82.

**Figure 7:**
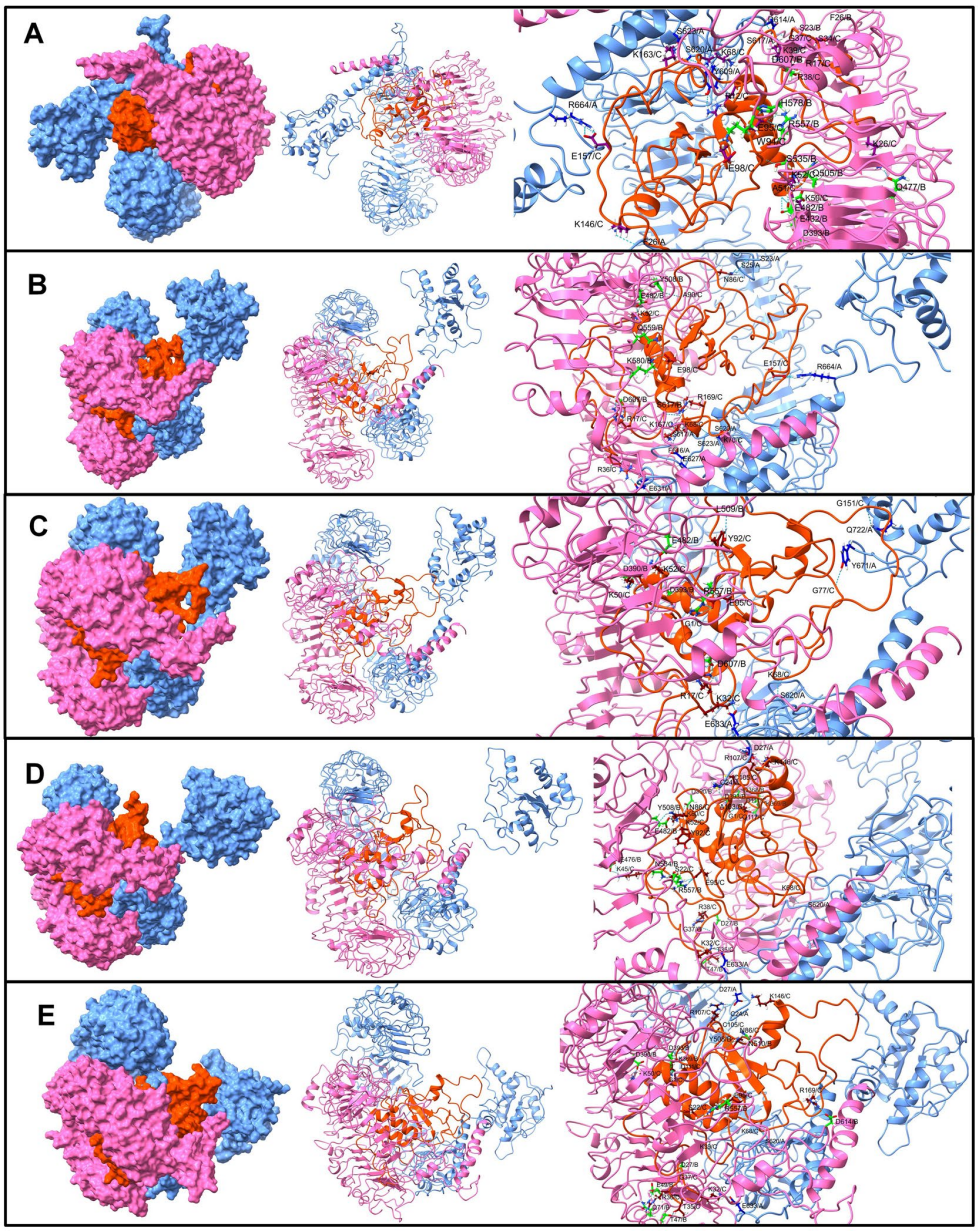
The inter-chain hydrogen bonds between TLR5 and vaccine-V1. (TLR5 chain A surface/cartoon is shown in light blue color, TLR5 chain B are in pink color, while the vaccine is in brown color).

The equilibrated trajectory of TLR5-V1 complex showed hydrogen bonds between vaccine-V1 residues Arg12, Arg17, Lys26, Gly37, Arg38, Lys39, Lys50, Ala51, Lys52, Lys68, Trp94, Lys146, Lys163, Glu157, Ser34, Glu95, and Glu98 and residues from TLR5 chain A Ser620, Arg664, Tyr609, Asp614, Ser617, Phe26, Ser623, and residues from chain B Phe26, Arg557, His578, Asp507, Gln477, Ser23, Asp393, Glu432, Glu482, Gln505, and Ser535 (Fig. 7). Out of these hydrogen bonds, the hydrogen bonds between vaccine-V1 residues Lys52 and Lys68, chain A residue Ser620 and chain B residue Glu482 remained intact until the end of the simulation. In addition to these stable hydrogen bonds, the trajectory isolated at 25 ns showed a hydrogen bond between vaccine-V1 residues Arg17, Arg36, Lys70, Asn86, Lys167, Arg169, Asn86, Glu157, and Glu98 and TLR5 chain A residues Ser25, Arg664, Glu631, Phe616, Ser617, Ser623, Ser23, Glu627, and chain B residues Tyr508, Gln559, Ls580, Asp607, and Ser617. Slightly fewer hydrogen bonds were observed in the 50 ns trajectory, where the vaccine-V1 residues Gly1, Arg17, Lys32, Arg36, Lys36, Lys50, Thr92, Gly77, Gly151, and Glu95 formed a hydrogen bond with TLR5 chain A residues Tyr671, Gln722, Glu633, and chain B residues Arg557, Asp393, Asp607, Thr47, Asp390, Glu482, and Leu509. Amongst these hydrogen bonds, the hydrogen bonds between vaccine-V1 residues Gly1, Lys32, Arg36, Lys50 and TLR5 chain A residue Glu633, and chain B residues Arg557, Asp393, Thr47, Asp390 remained stable until the end of the simulation. Few more stable hydrogen bonds between vaccine-V1 residues Asn86, Cys105, Arg107, Lys146, Gly37, Thr35, Asp111, Ser22, Glu95, and TLR5 chain A residues Cys24, and Asp27, and chain B residues Asp27, Lys369, and Thr508 were observed in 75 ns and 100 ns trajectories.

**Figure 8:**
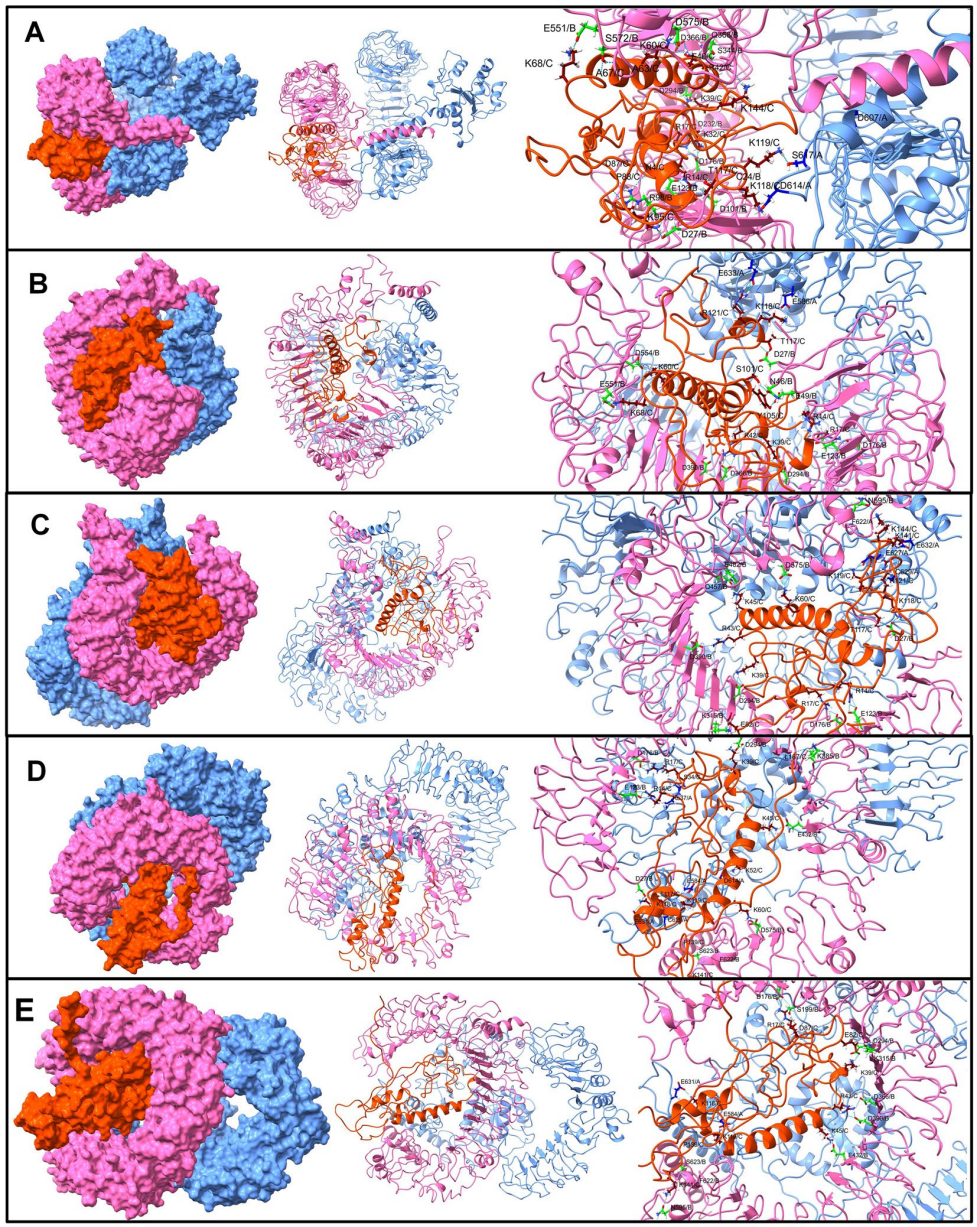
The inter-chain hydrogen bonds between TLR5 and vaccine-V2. (Similar color scheme as Fig. 8).

The equilibrated trajectory of TLR5-V2 showed the hydrogen bonds between the vaccine-V2 residues Arg14, Arg17, Lys32, Lys39, Arg42, Lys60, Ala67, Lys68, Lys95, Thr117, Lys118, Lys119, Asn4, Asp87, Pro88, Glu46, Ala63, and chain A residues Asp614, Ser617, and Asp607, and chain B residues Arg98, Gln368, Ser572, Glu123, Asp101, Asp176, Asp232, Asp27, Asp294, Ser344, Asp366, Asp575, Ser572, Glu551, and Cys24 (Fig. 8). The 25 ns trajectory showed that out of these hydrogen bonds the hydrogen bonds between vaccine-V2 residues Arg14, Lys39, Arg42, Lys60, Lys68, Thr117, and Lys118 and TLR5 chain B residues Glu123, Asp294, Asp366, Glu551, Asp27 remained stable and new hydrogen bonds between vaccine-V2 residues Arg17, Ser101, Thr105, Arg121, and TLR5 chain A residues Glu586, and Glu633, and chain B residues Asp176, Asp390, Asp554, Asn46, Glu49 were formed. Most of these hydrogen bonds remained stable until the end of the simulation period. However, the 50 ns trajectory showed new hydrogen bonds between vaccine-V2 residues Arg43, Lys45, Lys119, Lys141, Lys144, and Glu82, and TLR5 chain A residues Glu627, Glu632, and Phe622, and chain B residues Lys315, Asp390, Gln457, Glu432, Asn595. Out of these newly formed hydrogen bonds, the hydrogen bonds between vaccine-V2 residues Lys45, Lys199, and Lys141, and chain B residues Asp390 and Glu432 remained stable until the end of the simulation. Few new hydrogen bonds between vaccine-V2 residues Lys52, Ser34, Leu167, and Pro139, and chain A residues Arg537, Asp614, and chain B residues Lys385, Ser623, and Phe622 were observed in 75 ns trajectory. The 100 ns trajectory showed new hydrogen bonds between vaccine-V2 residues Arg43, Asp87, Glu82, and chain B residues Ser199, Lys315, Glu432, Phe622, and Asn595, in addition to the stable hydrogen bonds.

#### Contact map analysis

**Figure 9:**
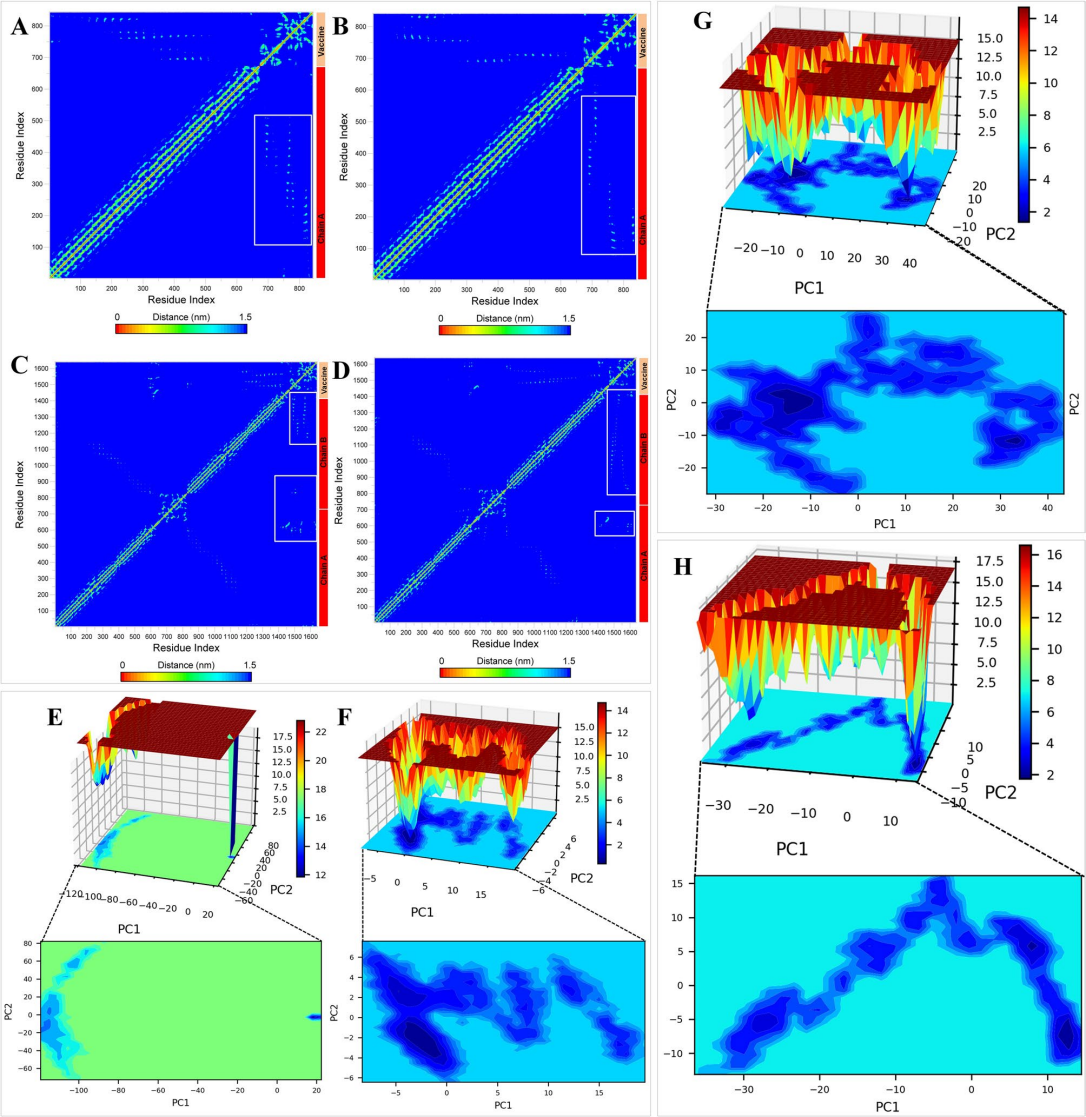
Contact maps constructed for the mean smallest distances between a network of residues of TLRs and vaccines. (**A**) TLR3-V1 complex, (**B**) TLR3-V2 complex, (**C**) TLR5-V1 complex, and (**D**) TLR5-V2 complex. (The key contacts are marked with white rectangles). Gibb’s free energy landscape. (**E**) TLR3-V1 complex, (**F**) TLR3-V2 complex, (**G**) TLR5-V1 complex, and (**H**) TLR5-V2 complex.

The contact analysis showed that the vaccine-V1 forms unique contacts with residues in the range of 100–500 from TLR3 (Fig. 9A–D). In this figure, the residue index between 0 and 700 represents the chain A residues 0–696, the residue index of 700–1400 represents the chain B residues 0–696, and the residue index above 1400 represents vaccine-V1 residues. The vaccine-V2 showed a slightly broader range of contacts with residues 100–600 from TLR3. In the case of the TLR5-V1 complex, the vaccine-V1 makes fewer contacts with chain A residues in the range 600–696. More residue-residue contacts were seen between vaccine-V1 and chain B residues 500–696 and a few from 100 to 200 residues. The TLR5-V2 complex showed a slightly broader range of contacts between vaccine-V2 and chain B residues from 100 to 696. Few residues of chain A in the range 550–696 also make residue-residue contacts with vaccine-V2.

#### Gibb’s free energy analysis

TLR3-V1 complex showed a small energy basin with energy − 100 to − 120 kJ mol^−1^ on PC1 and − 20 to − 40 kJ mol^−1^ on PC2, occupied by the lowest energy and stable conformations (Fig. 9E–H). TLR3-V2 complex, on the other hand, showed a larger energy basin with energy 0 to − 5 kJ mol^−1^ on PC1 and 0 to − 6 kJ mol^−1^ on PC2. Many other lower energy basins were observed for TLR3-V2 complex spread across the energy range 20 to − 10 and 7 to − 7 kJ mol^−1^ on PC1 and PC2, respectively. In the TLR5-V1 complex, the lowest energy conformations were observed in the energy basin with energy − 10 to − 20 kJ mol^−1^ on PC1 and 10 to − 10 kJ mol^−1^ on PC2. TLR-V1 complex also showed many other lower energy basins spread across the energy range 50 to − 30 and 30 to − 30 kJ mol^−1^ on PC1 and PC2, respectively. In the case of the TLR5-V2 complex, ample low-energy conformations were observed. However, the lowest energy conformations were found in the energy basin with energy 15–10 kJ mol^−1^ on PC1 and − 5 to − 10 kJ mol^−1^ on PC2.

#### Dynamic cross-correlation (DCC) analysis

The time-correlated information of inter-chain and intra-chain residue to residue contacts and motions was analyzed from the DCC matrix. Figure 8 shows the DCCM plots where the color gradient ranges from blue (negative correlation, less likely) to red (positive correlation, more evident), corresponding to the correlation coefficients − 1 and + 1, respectively. The lighter shades of these colors indicate weaker correlations, while the white color indicates no correlation. In the case of the TLR3-V1 complex, the DCC matrix showed that very few residues of TLR3, ranging from 200 to 400, have a weak positive correlation with vaccine-V1 residues (Supplementary Fig. S10). On the other hand, the vaccine-V2 showed more number of slightly stronger positive correlations with almost all TLR3 residues. In the case of the TLR5-V1 complex, vaccine-V1 residues showed a strong positive correlation with almost all the residues of TLR5 chain A residues and residues ranging from 400 to 600 from chain B. In the case of the TLR5-V2 complex, a slightly weaker positive correlation between vaccine-V2 residues and residues ranging from 100 to 696 from chain A and residues from 400 to 696 from chain B was observed.

#### DSSP analysis

It is evident from the DSSP plots that the TLR chains are reasonably stable with respect to the secondary structures (Supplementary Fig. S11). The vaccine-V1 showed more secondary structural changes when bound to TLR3 than when bound to TLR5. Here, the secondary structure is affected in the residues ranging from 15 to 50 and 60–169. Similarly, the vaccine-V2, when bound to TLR3, also showed more secondary structural changes than when bound to TLR5. The residues ranging from 1–30, 60–100, and 120–169 were seen affected in the secondary structure.

#### MM-PBSA calculation

The results of MM-PBSA calculations are given in Table 4. The vaccine-V2 has the lowest electrostatic energy and solvent accessible surface area (SASA) energy contribution with TLR5, where these energies are − 6631.372 and − 93.826 kJ mol^−1^, respectively. Vaccine-V2 also showed higher polar solvation energy of 2793.773 kJ mol^−1^ with TLR5. The binding free energy estimate for vaccine-V2 is − 4499.848 kJ mol^−1^, which is the lowest compared to its bound complex with TLR3 and compared to vaccine-V1 bound to both TLRs. Specifically, vaccine-V2 bound to TLR3 showed slightly higher electrostatic energy and SASA energy of -2310.507 and − 59.154 kJ mol^−1^, respectively. It also showed a comparably lower polar solvation energy of 1256.994 kJ mol^−1^ when bound to TLR3. Vaccine-V1 has comparably higher binding energy (ΔG_binding_) of − 1175.405 kJ mol^−1^ in complex with TLR3 than in complex with TLR5 with the binding energy of − 1975.729 kJ mol^−1^. The binding energy (ΔG_binding_) estimates for the trajectories sampled at each ns during the MD simulation are shown in (Supplementary Fig. S12). The binding energy remains almost constant in trajectories until 80 ns simulation with an average of around − 1400 kJ mol^−1^ and thereafter remains slightly higher during the remaining simulation period. In the TLR3-V2 complex, the binding energy fluctuates until 40 ns simulation and remains constant with an average of around − 1500 kJ mol^−1^. In the TLR5-V1 complex, after initial fluctuation until the first 15 ns, the simulation remains consistent at around − 2000 kJ mol^−1^. Interestingly, in the TLR5-V2 complex, the binding energy remained consistently lowest after the first 10 ns simulation period with an average of around − 4500 kJ mol^−1^.

**Table 4:** Results of MM-PBSA calculations.

Complex with vaccine	van der Waal energy (kJ mol^−1^)	Electrostatic energy (kJ mol^−1^)	Polar solvation energy (kJ mol^−1^)	Solvent accessible surface area energy (kJ mol^−1^)	Binding energy (ΔG_binding_) (kJ mol^−1^)
TLR3-V1	− 359.415 (16.586)	− 2105.109 (80.707)	1346.007 (64.923)	− 54.622 (2.659)	− 1175.405 (40.281)
TLR3-V2	− 414.088 (4.707)	− 2310.507 (12.856)	1256.994 (19.352)	− 59.154 (0.566)	− 1524.915 (16.130)
TLR5-V1	− 636.303 (16.658)	− 2982.461 (23.163)	1728.381 (41.431)	− 87.564 (2.053)	− 1975.729 (15.395)
TLR5-V2	− 574.375 (6.279)	− 6631.372 (37.729)	2793.773 (44.692)	− 93.826 (0.788)	− 4499.848 (28.268)

Standard deviations are given in parentheses.

### Codon adaptation and in-silico cloning studies

The *E. Coli* strain K12 was selected as the host organism for the cloning process of V1, the vaccine construct. During the reverse transcription of the vaccine protein, the Codon Adaptation Index (CAI) was determined to be 1.0, indicating a high level of codon optimization. Notably, the optimized codons exhibited a GC content of 55.26%. Furthermore, the vaccine construct was carefully designed to be free of ApaI and BgII restriction sites, ensuring the safety and integrity of the cloning process. The pET28a(+) vector, used for cloning, also contained ApaI and BglI restriction sites alongside the optimized codons. The resulting clone consisted of 4953 base pairs, with the desired sequence spanning 517 base pairs. The remaining base pairs originated from the vector. To emphasize the desired region, it was highlighted in red, as shown in Supplementary Fig. S13.

### Immune simulation studies

The immunological simulation study demonstrated that the immunisation can trigger a typical immune response commensurate with the natural immune system. The vaccine was expected to robustly activate primary immune responses after each of the three injections in sequence. Furthermore, the secondary immune response was activated, whereas the primary immune response gradually increased with each dose. Furthermore, following increases in the concentrations of active B cells, plasma B cells, helper T cells, and cytotoxic T cells were detected, indicating the establishment of an exceptionally robust immune response and memory of immunity, as well as increased antigen clearance in the host. The vaccine can produce many cytokines needed for immune response and virus defence, such as IFN-γ, IL-23, IL-10, and IFN-β. The results of the immunological simulation investigation are summarized and illustrated in Supplementary Fig. S14. In summary, immune simulation studies predicted a number of promising vaccine properties, such as the production of a large number of immunoglobulins, APCs, cytokines, and active B and T cells, implying that the polyvalent vaccine may generate exceptional immunological responses once administered to the host.

## Discussion

CyHV-3 and CyHV-1 belong to the Alloherpesviridae family and primarily infect fish and amphibians. CyHV-3 is an emerging pathogen responsible for severe diseases in common carp and koi fishes, while CyHV-1 causes a recurring skin disorder known as “carp pox” both causing significant economic losses in the global common and koi carp industries^9,18^. Developing an effective vaccine against these viruses is crucial for mitigating this severe problem in carp production worldwide. In this study, we for the first time developed two multi-epitope subunit vaccines targeting both CyHV-1 and CyHV-3 using a reverse vaccinology approach. Further molecular docking and molecular simulation dynamics revealed that both vaccines are promising candidates for preventing diseases in fish caused by CyHV-1 and CyHV-3. However, an in vivo laboratory and field trials are needed to validate the efficacies of these fish vaccines before recommending them for practical use in aquaculture against CyHV-1 and CyHV-3.

To design the vaccine, three outer membrane proteins with high antigenic scores were selected from each strain after conducting a comprehensive study of the entire proteome. From CyHV-1, we chose Membrane protein ORF25, Protein ORF136B, and Major Capsid Protein, while from CyHV-3, we selected Glycoprotein, Capsid triplex subunit 1, and ORF104 protein. These proteins demonstrated high antigenicity and were predicted to be located on the outer membrane, making them suitable targets for vaccine development.

While most vaccines primarily focus on B cell immunity, T cells play a crucial role in boosting the host's immune response against viral infections. Therefore, we predicted both T cell and B cell epitopes for vaccine development. T cell epitopes were predicted for each selected protein using the NetCTL prediction server. Among the predicted epitopes, the top four were selected based on the stringent criteria of high antigenicity, non-allergenecity and non-toxicity. These epitopes were evaluated for their binding affinity to HLA-A*1101 and HLA-DRB1*0401 alleles, which are two of the most prevalent alleles. Based on their binding affinity, the top six T-cell epitopes were selected for vaccine construction. The chosen epitopes demonstrated high antigenicity and strong binding energy with both the MHC-I and MHC-II alleles, making them excellent candidates for the constructed vaccine. To predict B cell epitopes, we employed six different epitope prediction approaches to mitigate false positive result. After analyzing the results and considering the antigenicity, allergenecity, and toxicity of the epitopes using several servers, we selected the 12 best B cell epitopes (two epitopes for each protein). All the chosen epitopes exhibited high antigenicity, non-toxicity, and non-allergenecity.

Consequently, a total of six T cell epitopes and 12 B cell epitopes were selected for constructing the vaccine. These epitopes underwent rigorous screening to ensure their compatibility and effectiveness in eliciting an immune response. To create a multi-epitope-based vaccination design, the predicted T cell and B cell epitopes were joined together using various linkers (EAAAK, AAY, and GPGPG) to ensure proper spatial separation. Addition of the EAAAK linker at the beginning or terminal end of the vaccine construct was specifically done to minimize the chances of vaccine degradation. The presence of AAY and GPGPG linker ensures the stability of respective epitopes. The inclusion of beta-defensin-3 adjuvants improved the antigenicity, immunogenicity, stability, and lifespan of the developed vaccines. Additionally, the vaccination designs incorporated the PADRE sequence, known for its strong immune-stimulating properties^83^.

Two vaccines were constructed using the selected epitopes. The safety and effectiveness of the vaccine formulations were assessed through several evaluations. Vaccine V1 and V2 exhibited antigenicity scores of 0.9457 and 0.8430, respectively, indicating their ability to stimulate an immune response. Furthermore, both vaccines were found to be non-allergenic and non-toxic. In terms of solubility, V2 exhibited higher solubility than V1, as determined by the Protein-Sol server. The stability indices for V1 and V2 indicated their robustness, as scores below 40 indicate stability. Additionally, the negative GRAVY values of V1 and V2 indicated their suitability as vaccine candidates. A negative GRAVY value suggests hydrophilic properties, implying that the components will dissolve easily in water. The aliphatic index (AI) value, which evaluates thermostability, was 48.70 and 63.29 for V1 and V2 respectively, suggesting that the vaccine was stable within normal body temperature as it is higher than normal body temperature of fish.

Tertiary structure predictions were performed for both vaccine constructs. The best tertiary model for Vaccine V1 achieved an ERRAT score of 76.875, with 74.8% of the structure falling within the favoured region while Vaccine V2 obtained an ERRAT score of 75.4 with 65.9% within the favoured region based on the Ramachandran plot. The Z score graphs for both constructs indicated their appropriateness and quality.

Molecular docking was performed for both vaccines with TLR3 and TLR5. The docking was carried out with Cluspro server, which uses an algorithm for the effective docking of peptide motifs to their free receptor structures^84^. One of the interesting findings is that both vaccines exhibited strong affinity toward TLR5. TLR5 is commonly utilized in fish vaccine construction, while TLR3 plays a significant role in viral infections. TLR3 has been discovered in various fish species, including channel catfish. Infections with the *C. herpesvirus* are also associated with TLR3 upregulation^85,86^. In docking analysis, V2 demonstrated the highest affinity with the lowest energy score of -1331.8, while V1 also displayed a high binding affinity with a docking energy of -1226.9. Consequently, both vaccines have the potential to activate TLRs and enhance the immune response against the viruses. Molecular dynamics simulations were performed for both the vaccine complexes with TLR3 and TLR5 to further investigate their behaviour and stability.

TLR3 has a single chain, while TLR5 has two chains, chain A and chain B. The vaccine constructs V1 and V2 are bound to TLR3, occupying the non-terminal site. In contrast, vaccine constructs bind at the interface of chain A and chain B in TLR5. Such unique binding of vaccine constructs might impose conformational restrictions on the residues close to vaccine constructs. It is evident from the results of RMSD that TLR5 chain A undergoes a substantial conformational change during simulation from the initial equilibrated positions. While the TLR5 chain B has restricted flexibility and minor conformational changes occurred in it. Comparably TLR3 has lesser deviations than TLR5 chain A or chain B. These results suggest that vaccine constructs stabilize the TLR5 chain B better than chain A, and vaccine construct-V1 is better in stabilizing TLR3 than vaccine-V2. These results were further confirmed by the RMSD in vaccines where V1 has the slightest deviations suggesting its good stability when bound to TLR5.

Another important finding is that vaccine-V1 was stable with minimal deviations from the equilibrium state. The RMSD in vaccine-V2 is stable until around 70 ns and a magnitude of 0.5 nm deviations occurred thereafter. The binding energy also corroborates the results that vaccine-V2 is stable with the lowest binding energy and deviates slightly thereafter.

The RMSF analysis also confirms that vaccines bound to TLR3 and TLR5 chain B have minimal fluctuations. While chain A of TLR5, which is not in close contact with vaccine constructs, showed larger fluctuations. The fluctuations in vaccine-V2 bound to TLR3 are maximal, suggesting that it undergoes more conformational changes than the other corresponding systems.

The analysis of the radius of gyration gives an insight into the system's compactness. The results of RMSD and RMSF further corroborate that chain A of TLR5 undergoes major conformational changes and, thus, is the least compact. However, the vaccine-V1 bound to TLR5 showed the lowest magnitude of gyration radius and suggested a compact and stable structure. It was found that chain B of TLR5, which is in close contact with vaccine-V1, also has a reasonably compact and stable structure which suggests stable interaction of vaccine-V1 to chain B of TLR5 chain B. Similarly, TLR5 bound to vaccine-V1 shows reasonably compact and stable vaccine-V1 structure and chain B of TLR5. In the case of TLR3 complexes, both vaccine-V1 and vaccine-V2 have stable and compact structures along with stable and compact structures of TLR3.

The more the number of interchain hydrogen bonds, the more the binding affinity between the corresponding systems will be. Amongst all the studied systems, TLR5 chain B and vaccine-V2 showed more hydrogen bonds, and around 13 consistent hydrogen bonds were formed. Further, only chain B of TLR5 seems to form more hydrogen bonds than chain A. Thus, both the vaccine constructs stabilize the chain B of TLR5 and might have a very strong affinity for stabilizing TLR in turn. TLR3 also formed a reasonably good number of hydrogen bonds for both vaccine constructs, but fewer in number than TLR5, suggesting a slightly less binding affinity between vaccine constructs and TLR3 than TLR5. The analysis of a few stable hydrogen bonds between vaccine residues and TLR5 chain B residues further confirms the strong affinity between the vaccines and TLR5.

The contact analysis also corroborates the hydrogen bond analysis results where the TLR2-vaccine complex has more residue-residue contacts between chain C and chain D of TLR2 and the vaccine chain. On the other hand, fewer residues of TLR4 chains B and D could establish such contacts with vaccine residues.

Gibb’s energy analysis confirmed the larger number of conformations with the lowest energy below − 20 kJ mol^−1^ on PC1 and − 5 kJ mol^−1^ in the case of the TLR5-V2 complex. TLR3-V1 complex also showed the lowest energy conformations, but fewer conformations exist in the lowest energy basin below − 100 kJ mol^−1^ on PC1 and around − 20 kJ mol^−1^ on PC2.

The DCC analysis revealed that the TLR5-V1 complex has more prominent strong, positively correlated residue-residue cross-walks than other complexes. TLR5-V2 complex also showed better residue-residue crosswalks than TLR3-vaccine complexes. The results of the DCC analysis confirm the stability of TLR5-vaccine complexes.

The DSSP analysis suggests the stable secondary structures of TLR chains. Especially, TLR3 and TLR5 chain B showed minimal secondary structural changes suggesting stable conformations of these TLRs. The vaccine constructs having a larger proportion of loops showed a number of secondary structural changes in different regions. However, the vaccine constructs, V1 and V2, bound to TLR5, showed fewer secondary structural changes. This confirms the stable interactions of vaccine constructs with TLR5.

The MM-PBSA analysis suggested a better than two-fold affinity of vaccine-V2 to TLR5 than TLR3. The vaccine-V1 has a somewhat better affinity to TLR5 than TLR3. The better binding affinity of vaccine-V2 is due to the lowest electrostatic, SASA energy and higher polar solvation energy. The efficiency of the vaccine's translation was predicted by enhancing the mRNA using the Java Codon Adaptation tool, with the E. coli strain K-12 serving as the cell culture system. The resulting clone consisted of 4953 base pairs, with 517 base pairs corresponding to the desired sequence and the remaining base pairs derived from the vector. This cloned vector holds promise for heterologous cloning and expression of the vaccine.

The production of species-specific vaccines necessitates a thorough understanding of fish immune systems, which include B-cells, T-cells, MHC molecules, and TLR signalling pathways; nevertheless, standard methods are frequently expensive and time-consuming. Immunoinformatics is a promising option for more effective vaccine design, capable of dealing with the difficulties of emerging and re-emerging diseases, antigenic diversity, and personalised immunisation needs. This technique uses high-performance tools to identify multi-epitope vaccines, offering a platform for studying differences in immune adaptations among fish species^87^. Compared to traditional vaccines, which may revert to a harmful form or not undergo complete inactivation, recombinant subunit vaccines are considered highly safe. This is because they only contain antigenic components, rather than the entire virus, eliminating the risk of developing harmful traits. The current study confirmed that the subunit vaccines constructed in this manner are non-toxic and non-allergenic. To further assess the potential of our developed vaccines, further additional experimental studies in a laboratory setting using model animals are recommended. The study could pave the way for future rapid vaccine development against *C. herpesvirus* and also other fish pathogen.

## Conclusion

All around the world, *C. herpesviruses* 1 and 3 play a significant threat to aquaculture. We developed two multi-epitope subunit vaccines targeting both CyHV-1 and CyHV-3 using immunoinformatics approach. Our method will enable quick prediction of vaccination candidates' potential prior to experimental testing. Conducting in vivo and in vitro experiments against these ecologically significant viruses will be made more straightforward by our *in-silico* investigation.

### Supplementary Information

Supplementary Information.

## Data Availability

All the data acquired throughout the study are included in the manuscript/supplementary material. For further information, do contact the corresponding author.
