# Supplementary material for: Development of multi epitope subunit vaccines against emerging carp viruses Cyprinid herpesvirus 1 and 3 using immunoinformatics approach
Source: Sci Rep. 2024 May 23;14:11783. doi: 10.1038/s41598-024-61074-7 (PMC11116410; doi:10.1038/s41598-024-61074-7)
Supplement: Supplementary file 1 — Supplementary Information. [file 41598_2024_61074_MOESM1_ESM.docx]

**Immunoinformatics approach to develop a multi epitope subunit vaccine against emerging carp viruses Cyprinid herpesvirus 1 & 3**

Nurul Amin Rani^a, 1^, Tanjin Barketullah Robin^a, 1^, Anindita Ash Prome^a,^ ^1^, Nadim Ahmed^a, 1^, Abu Tayab Moin^b, 1^, Rajesh B. Patil^c, 2^ , Mohammad Nurul Azim Sikder^d, 2^, Md Nazmul Islam Bappy^a,e 2,^ Dilruba Afrin^a,e^, Ferdaus Mohd Altaf Hossain ^f,g^ , Tofazzal Islam* ^h^ , Kazi Md. Ali Zinnah* ^a,e^

**Supplementary Information**

**Tables**

Supplementary Table S1: Tools and servers used in epitope prediction with their specific application, input and output.

| **Tool name** | **Application** | **input** | **output** |
| --- | --- | --- | --- |
| *VaxiJen v2.0* | Determine the antigenicity | Amino acid sequence  (Epitope/Protein/Vaccine) | Antigenic score |
| *TMHMM 2.0* | Determine the transmembrane topology | Amino acid sequence  (Epitope/Protein/Vaccine) | Location of segments (inside/outside) |
| *AllergenFP* | Determine the Allergenecity | Amino acid sequence  (Epitope/Protein/Vaccine) | Allergen/Non-allergen |
| *AllerTOP* | Determine the Allergenecity | Amino acid sequence  (Epitope/Protein/Vaccine) | Allergen/Non-allergen |
| *AlgPred2.0* | Determine the Allergenecity | Amino acid sequence  (Epitope/Protein/Vaccine) | Allergen/Non-allergen |
| *ToxinPred* | Determine the toxicity | Amino acid sequence  (Epitope/Protein/Vaccine) | Toxic/Nontoxic |
| *IFNepitope* | Determine the capacity of to induce IFN-γ | Amino acid sequence  (Epitope) | Inducer/Non-inducer |
| *IL4pred* | Determine the capacity of to induce IL-4 | Amino acid sequence  (Epitope) | Inducer/Non-inducer |
| *IL10pred* | Determine the capacity of to induce IL-10 | Amino acid sequence  (Epitope) | Inducer/Non-inducer |
| *IEDB epitope conservancy tool* | Determine the conservancy among targeted strain | Amino acid sequence  (Epitope) | Conservancy (%) |

**Supplementary Table S2:** Selected proteins with their Uniprot accession ID, antigenicity and transmembrane topology

| **Strain** | **Protein** | **Accession ID** | **Antigenicity** | **Topology** |
| --- | --- | --- | --- | --- |
| CyHV-1 | Membrane protein ORF25 | K7PBK6 | 0.5231 | Outside |
|  | Protein ORF136B | K7PCA0 | 0.5763 | Outside |
|  | Major Capsid Protein | Q52UN7 | 0.4994 | Outside |
| CyHV-3 | Glycoprotein | K9JF86 | 0.5292 | Outside |
|  | Capsid triplex subunit 1 | A3QMN4 | 0.4425 | Outside |
|  | Protein ORF104 | A3QMS2 | 0.5534 | Outside |

**Supplementary Table S3:** Selected T cell epitopes of Cyprinid herpesvirus 1 and Cyprinid herpesvirus 3 with their respective allergenecity, antigenicity, topology and toxicity

| **Virus** | **Protein** | **Epitope** | **Topology** | **AllerTop** | **AllergenFP** | **AllergenOnline** | **AllerMatch** | **Toxicity** | **Antigenic Score** |
| --- | --- | --- | --- | --- | --- | --- | --- | --- | --- |
| **CyHV-1** | **Major capsid protein** | KDLDLGELE | Outside | NA | NA | NA | NA | NA | 2.8750 |
|  |  | TKDLDLGEL | Outside | NA | NA | NA | NA | NA | 2.4159 |
|  |  | PEARDSEYG | Outside | NA | NA | NA | NA | NA | 2.2265 |
|  |  | YVGLGLMSK | Outside | NA | NA | NA | NA | NA | 2.1667 |
|  | **Protein ORF136B** | VLLVSVKAT | Outside | NA | NA | NA | NA | NA | 1.5455 |
|  |  | HVEVSVECG | Outside | NA | NA | NA | NA | NA | 1.4627 |
|  |  | SVVLLVSVK | Outside | NA | NA | NA | NA | NA | 1.1772 |
|  |  | VVLLVSVKA | Outside | NA | NA | NA | NA | NA | 1.1085 |
|  | **Membrane protein ORF25** | CPFKPLEWC | Outside | NA | NA | NA | NA | NA | 2.0065 |
|  |  | PFKPLEWCS | Outside | NA | NA | NA | NA | NA | 1.8029 |
|  |  | FKPLEWCSG | Outside | NA | NA | NA | NA | NA | 1.4965 |
|  |  | SLGGGGATR | Outside | NA | NA | NA | NA | NA | 1.4546 |
| **CyHV-3** | **Protein ORF104** | LTFKPNWQP | Outside | NA | NA | NA | NA | NA | 2.4678 |
|  |  | DVKLSDFSL | Outside | NA | NA | NA | NA | NA | 2.4450 |
|  |  | VKLSDFSLT | Outside | NA | NA | NA | NA | NA | 2.4300 |
|  |  | PLSIELTPM | Outside | NA | NA | NA | NA | NA | 2.3909 |
|  | **Glycoprotein** | LDYKDFALK | Outside | NA | NA | NA | NA | NA | 1.9904 |
|  |  | LLDYKDFAL | Outside | NA | NA | NA | NA | NA | 1.9330 |
|  |  | DQRSLLCLG | Outside | NA | NA | NA | NA | NA | 1.6188 |
|  |  | LLLDYKDFA | Outside | NA | NA | NA | NA | NA | 1.5263 |
|  | **Capsid triplex subunit 1** | LEPAWVDPR | Outside | NA | NA | NA | NA | NA | 2.1077 |
|  |  | TWENMEFSY | Outside | NA | NA | NA | NA | NA | 1.8863 |
|  |  | RAEPYPAEL | Outside | NA | NA | NA | NA | NA | 1.6423 |
|  |  | PIRAEPYPA | Outside | NA | NA | NA | NA | NA | 1.5821 |

**Supplementary Table S4:** Docking result of T cell epitopes against HLA-A*1101 and HLA-DRB-1*0401

| **Protein** | **Epitope** | **Docking Score** | **RMSD** |
| --- | --- | --- | --- |
| HLA-A*1101 | KDLDLGELE | -114.04 | 13.48 |
|  | TKDLDLGEL | -131.36 | 24.73 |
|  | CPFKPLEWC | -184.20 | 48.52 |
|  | PFKPLEWCS | -178.24 | 23.26 |
|  | HVEVSVECG | -142.70 | 54.52 |
|  | VLLVSVKAT | -174.21 | 54.40 |
|  | FKPLEWCSG | -214.50 | 49.55 |
|  | SLGGGGATR | -153.62 | 27.04 |
|  | SVVLLVSVK | -160.52 | 53.50 |
|  | VVLLVSVKA | -172.65 | 50.96 |
|  | PEARDSEYG | -167.45 | 50.50 |
|  | YVGLGLMSK | -167.54 | 50.09 |
|  | RAEPYPAEL | -154.35 | 46.54 |
|  | PIRAEPYPA | -202.33 | 51.01 |
|  | DQRSLLCLG | -168.93 | 23.87 |
|  | LLLDYKDFA | -162.49 | 53.92 |
|  | VKLSDFSLT | -186.77 | 50.79 |
|  | PLSIELTPM | -166.53 | 25.41 |
|  | LEPAWVDPR | -178.31 | 11.92 |
|  | TWENMEFSY | -190.98 | 48.86 |
|  | LDYKDFALK | -163.96 | 22.63 |
|  | LLDYKDFAL | -187.10 | 49.67 |
|  | DVKLSDFSL | -159.77 | 46.85 |
|  | LTFKPNWQP | -198.30 | 51.25 |
|  |  |  |  |
| HLA-DRB-1*0401 | KDLDLGELE | -128.39 | 42.81 |
|  | TKDLDLGEL | -139.82 | 71.05 |
|  | CPFKPLEWC | -207.45 | 46.87 |
|  | PFKPLEWCS | -209.54 | 45.95 |
|  | HVEVSVECG | -150.85 | 53.20 |
|  | VLLVSVKAT | -165.82 | 56.28 |
|  | FKPLEWCSG | -201.98 | 50.45 |
|  | SLGGGGATR | -153.12 | 79.91 |
|  | SVVLLVSVK | -172.87 | 49.75 |
|  | VVLLVSVKA | -186.63 | 50.86 |
|  | PEARDSEYG | -158.29 | 48.21 |
|  | YVGLGLMSK | -196.70 | 49.00 |
|  | RAEPYPAEL | -160.71 | 64.15 |
|  | PIRAEPYPA | -176.09 | 48.51 |
|  | DQRSLLCLG | -169.07 | 47.66 |
|  | LLLDYKDFA | -187.34 | 49.48 |
|  | VKLSDFSLT | -168.69 | 55.38 |
|  | PLSIELTPM | -172.32 | 19.23 |
|  | LEPAWVDPR | -183.90 | 49.90 |
|  | TWENMEFSY | -212.82 | 50.03 |
|  | LDYKDFALK | -178.53 | 33.83 |
|  | LLDYKDFAL | -180.11 | 83.03 |
|  | DVKLSDFSL | -156.24 | 79.19 |
|  | LTFKPNWQP | -217.80 | 46.30 |

**Supplementary Table S5:** Selected B cell epitopes with their respective antigenicity, transmembrane topology, allergenecity, and toxicity.

| **Strain Name** | **Protein name** | **Epitope** | **Antigenic Score** | **Allergenecity** | **Toxicity** | **Topology** |
| --- | --- | --- | --- | --- | --- | --- |
| CyHV-3 | Capsid triplex | CTRDGED | 1.3241 | NA | NT | Outside |
|  |  | YLVLAAL | 0.8631 | NA | NT | Outside |
|  | Glycoprotein | QRSLLCL | 1.6847 | NA | NT | Outside |
|  |  | TKKRRT | 1.4580 | NA | NT | Outside |
|  | Protein ORF 104 | PKKRRR | 2.4479 | NA | NT | Outside |
|  |  | KKRRRS | 2.1671 | NA | NT | Outside |
| CyHV-1 | Orf 25 | LTDLQTK | 1.3096 | NA | NT | Outside |
|  |  | PTKDPK | 1.2809 | NA | NT | Outside |
|  | orf 136B | SRQKNE | 1.3482 | NA | NT | Outside |
|  |  | KTNNRE | 1.1915 | NA | NT | Outside |
|  | Major capsid | KDTDKSR | 1.0480 | NA | NT | Outside |
|  |  | AVVPLLL | 0.5663 | NA | NT | Outside |

**Supplementary Table S6**: Antigenicity, allergenicity and biophysical properties of V1 and V2

| **Features** | **V1** | **V2** |
| --- | --- | --- |
| **Antigenicity** | 0.9457 (Probable ANTIGEN ). | 0.8430 (Probable ANTIGEN ). |
| **Allergenicity** | Probable non-allergen (AllerTOP v.2.0)  Probable non-allergen (AllergenFP v.1.0) | Probable non-allergen (AllerTOP v.2.0)  Probable non-allergen (AllergenFP v.1.0) |
| **Solubility** | 0.773 | 0.793 |
| **Number of amino acids** | 169 | 167 |
| **Theoretical Isoelectric point (pI)** | 9.79 | 10.07 |
| **Formula** | C_801_H_1275_N_243_O_230_S_10_ | C_800_H_1287_N_237_O_210_S_9_ |
| **Total number of atoms** | 2559 | 2543 |
| **(Asp + Glu)** | 14 | 10 |
| **(Arg + Lys)** | 32 | 33 |
| **Half-life** | 30 hours | 30 hours |
| **Aliphatic index** | 48.70 | 63.29 |
| **Instability index** | 35.95 (Stable) | 35.12 (Stable) |
| **GRAVY** | -0.802 | -0.506 |

**Supplementary Table S7:** Population coverage analysis

| **Region** | **Population coverage on average** | **Average hit** |
| --- | --- | --- |
| World | 83% | 3.29 |
| East Asia | 82.91 | 3.2 |
| North Asia | 74.29% | 2.85 |
| South Asia | 70.67% | 2.68 |
| Europe | 88.91% | 3.64 |
| North America | 79.7% | 3.05 |

**Figures**

**
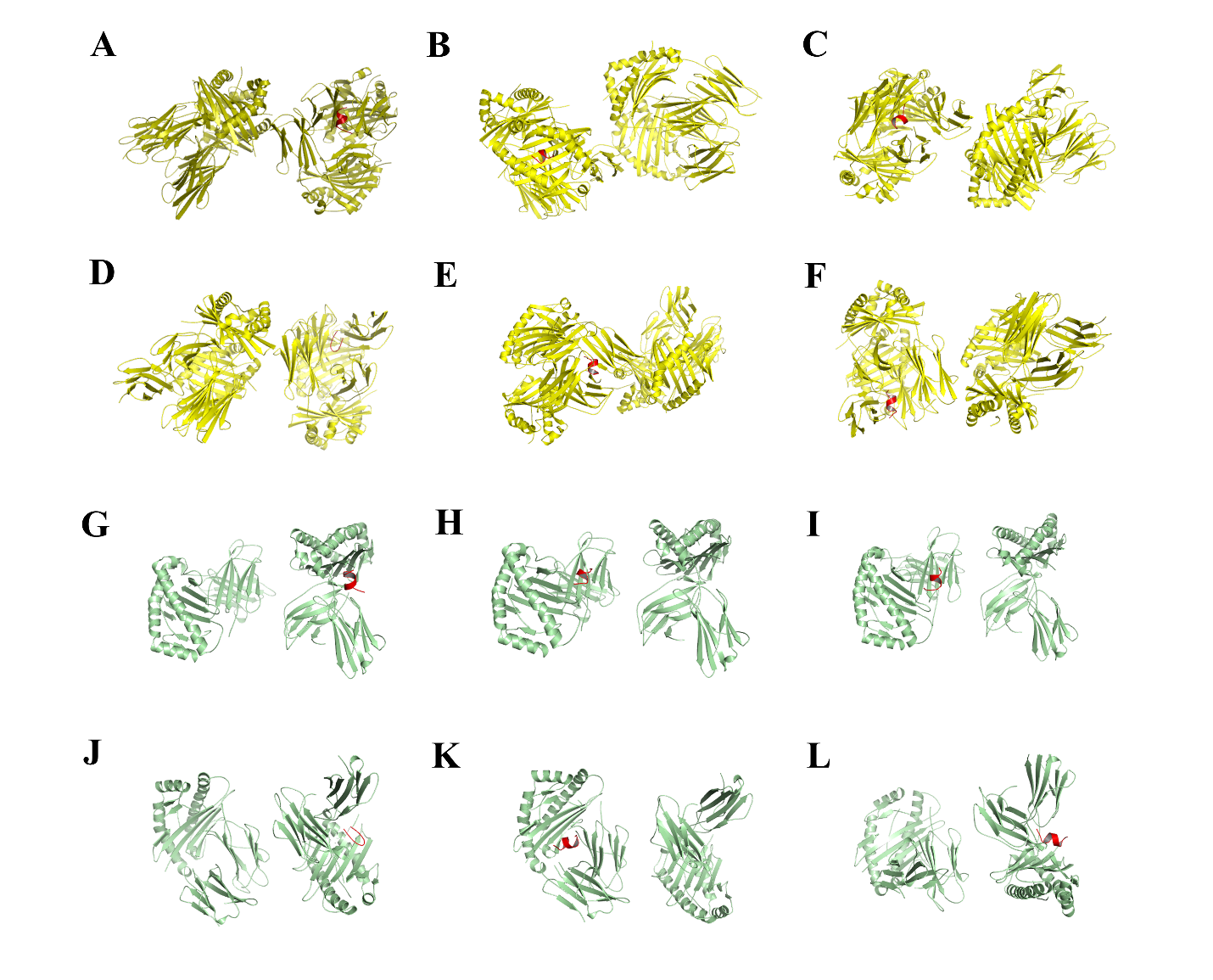
**

**Supplementary Figure S1:** Docking complex of MHC alleles with T cell epitopes (A) HLA-DRB-1*0401 & CPFKPLEWC (B) HLA-DRB-1*0401 & FKPLEWCSG (C) HLA-DRB-1*0401 & LEPAWVDPR (D) HLA-DRB-1*0401 & LTFKPNWQP (E) HLA-DRB-1*0401 & PFKPLEWCS (F) HLA-DRB-1*0401 & TWENMEFSY (G) HLA-A*1101 & CPFKPLEWC (H) HLA-A*1101 & FKPLEWCSG (I) HLA-A*1101 & LEPAWVDPR (J) HLA-A*1101 & LTFKPNWQP (K) HLA-A*1101 & PFKPLEWCS (L) HLA-A*1101 & TWENMEFSY

**
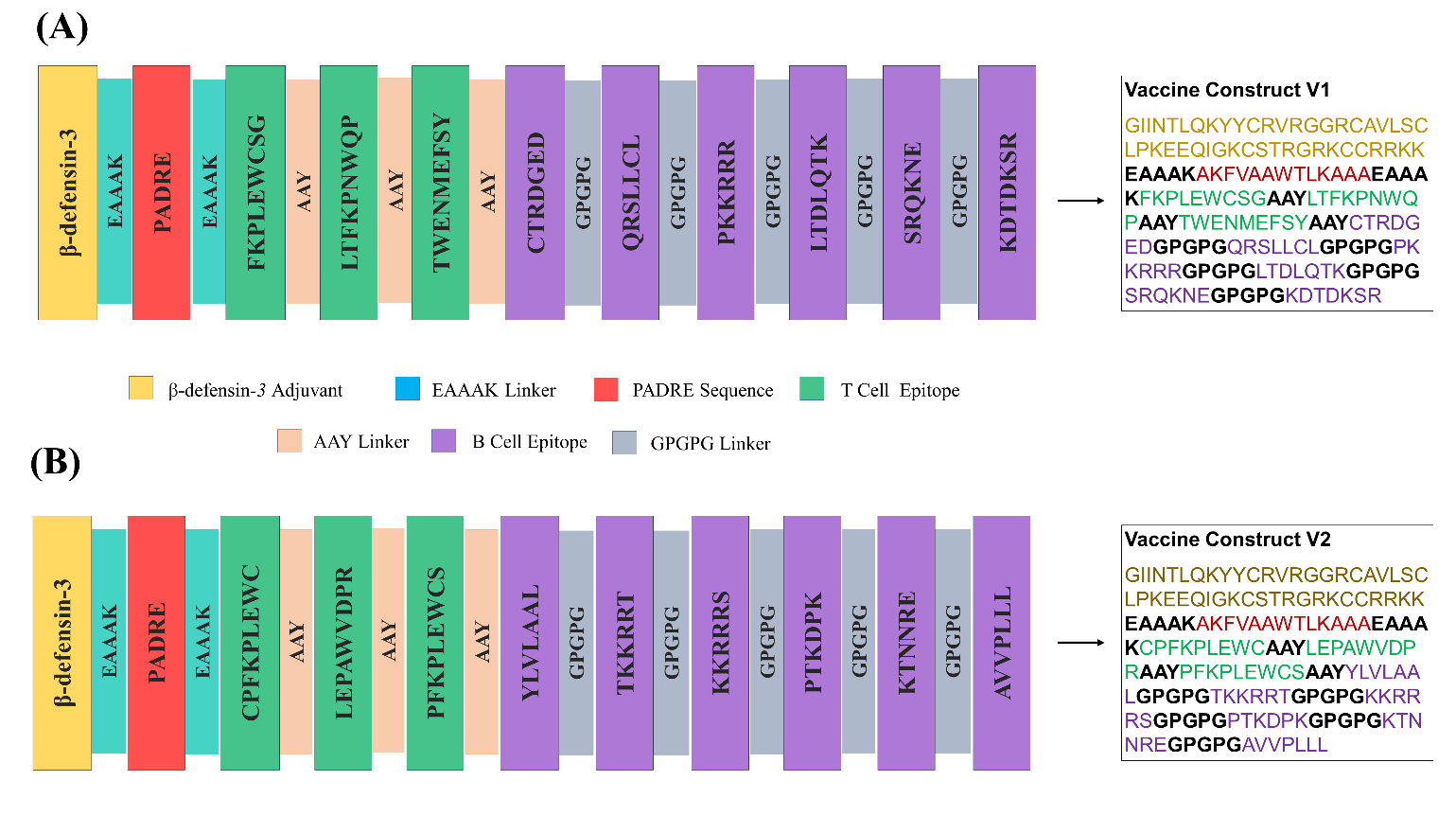
**

**Supplementary Figure S2:** Schematic and constructive diagram of (A) vaccine V1 and (B) vaccine V2


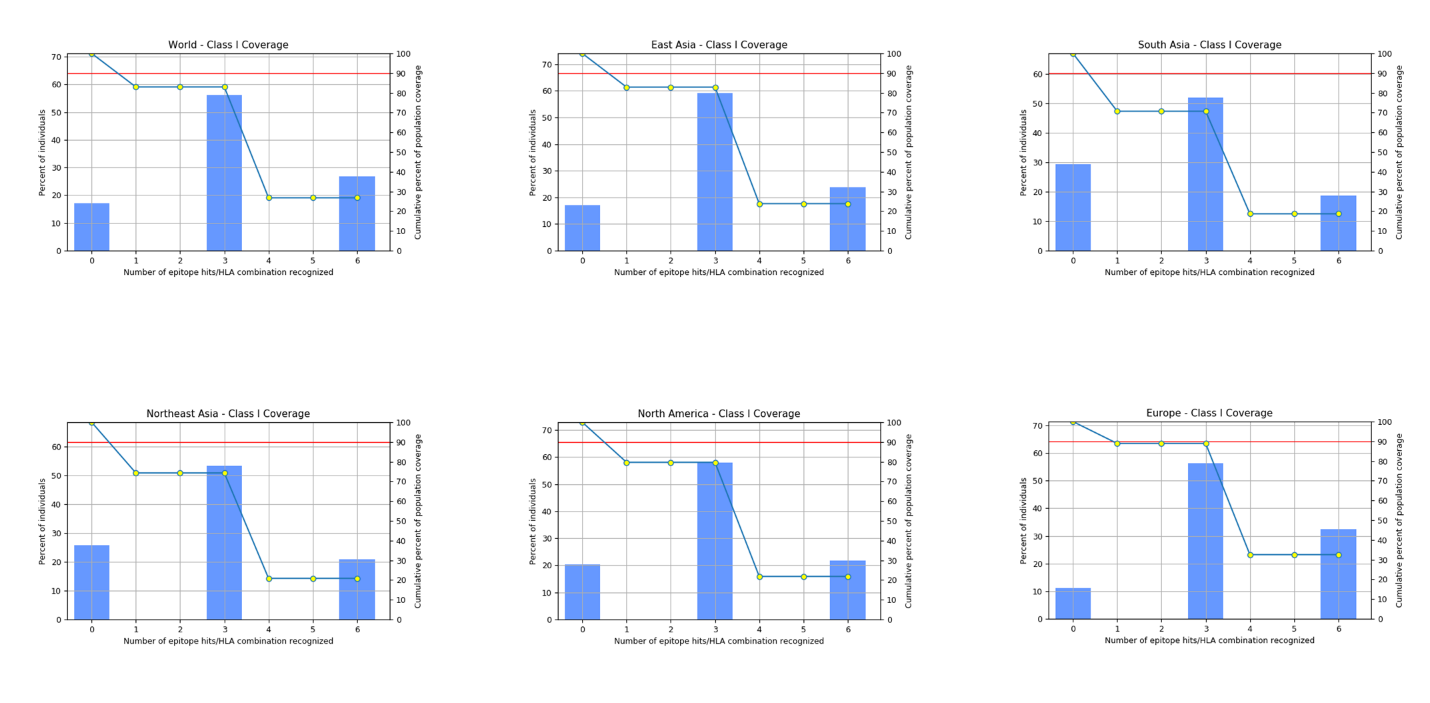


**Supplementary Figure S3:** Population coverage analysis graph of different region for the developed vaccine


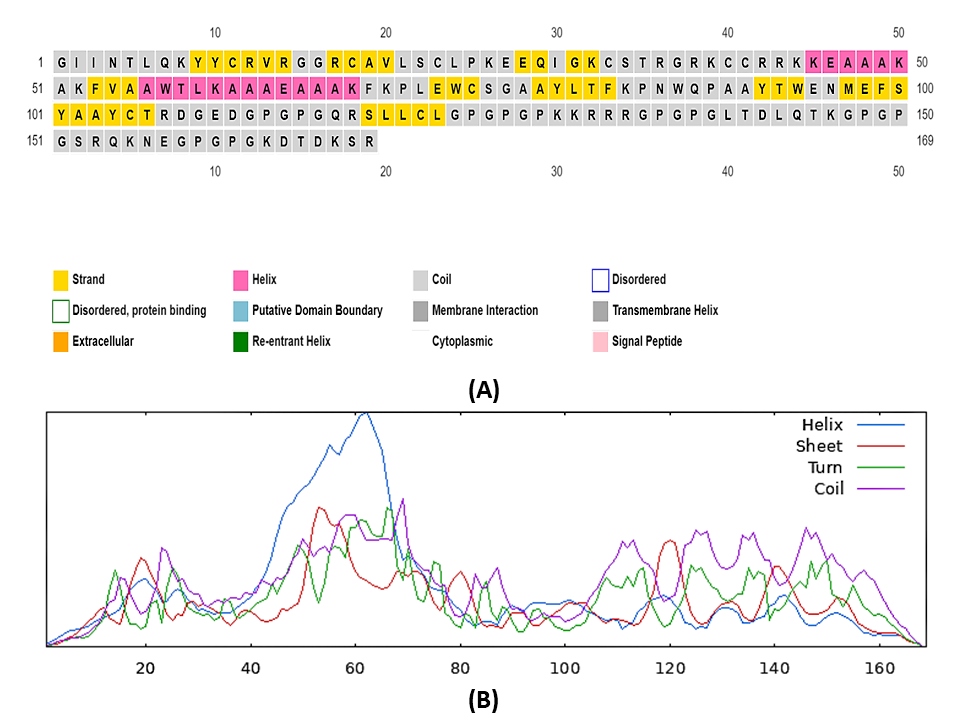


**Supplementary Figure S4:** Secondary Structure analysis of V1 (A) Generated by PsiPred (B) Helix, sheet, turn and coil analysis generated by SOPMA


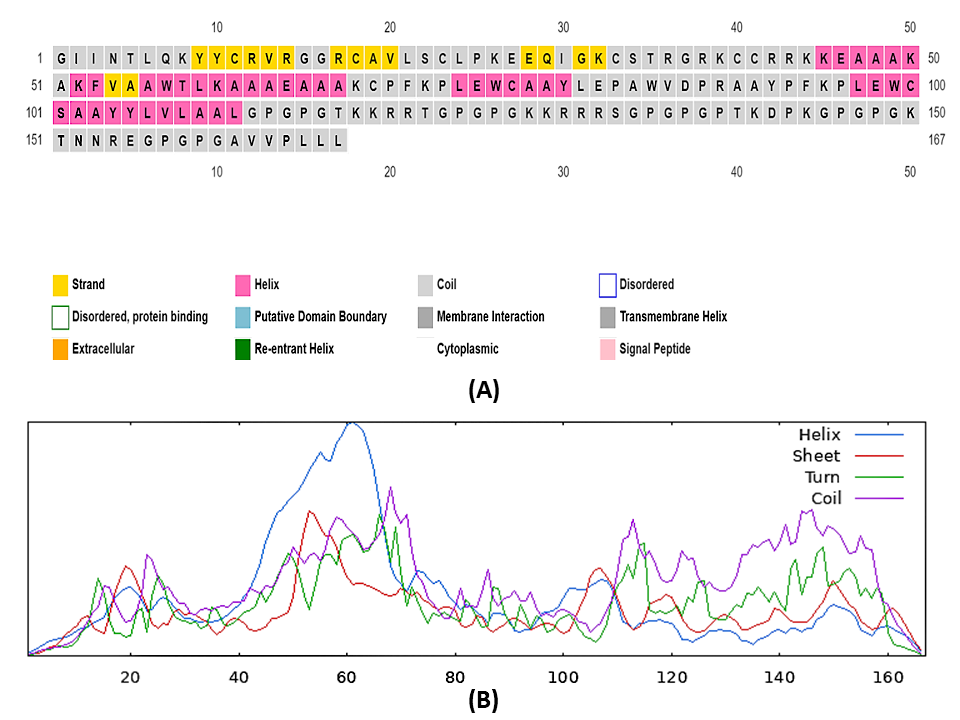


**Supplementary Figure S5:** Secondary Structure analysis of V2 (A) Generated by PsiPred (B) Helix, sheet, turn and coil analysis generated by SOPMA


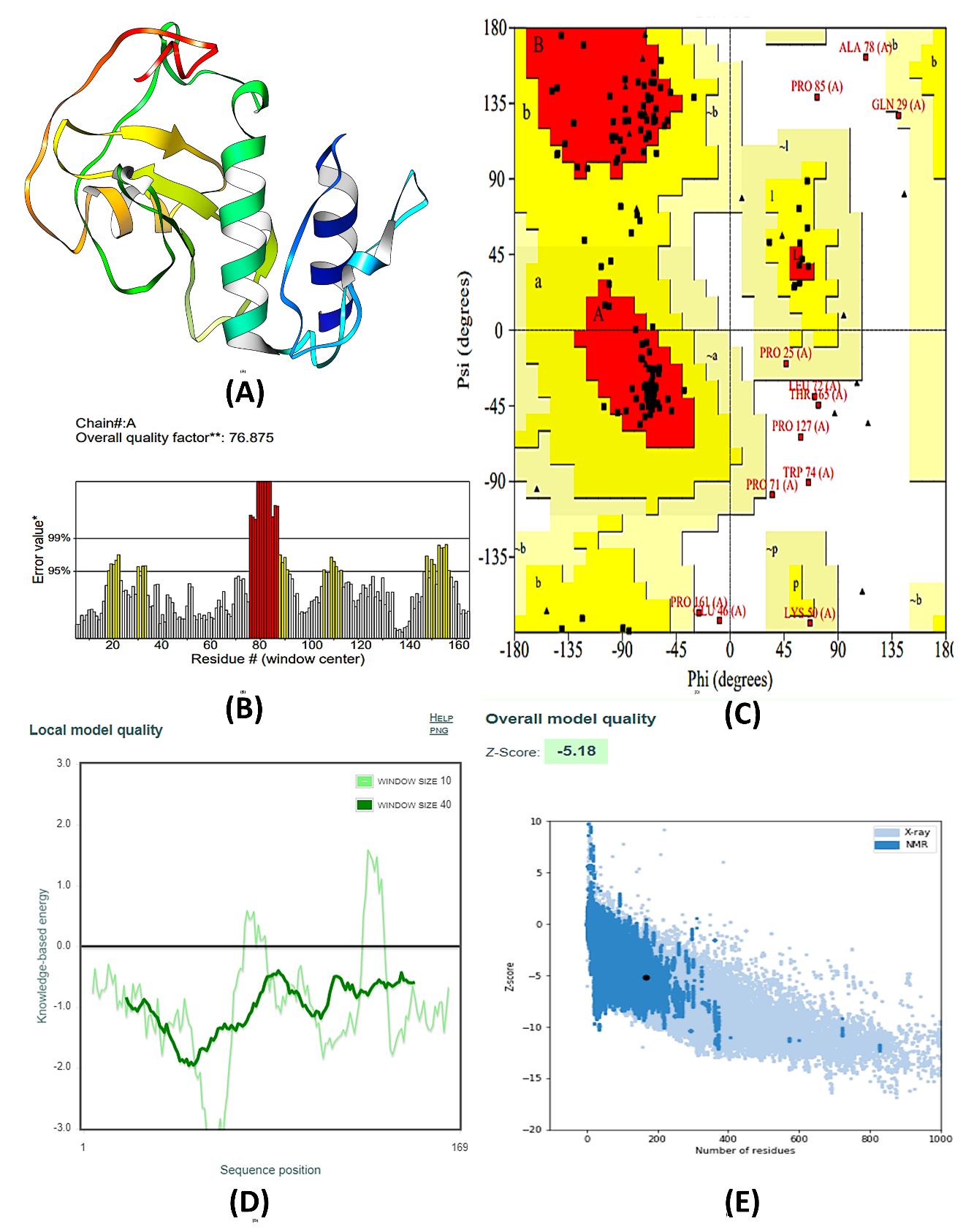


**Supplementary Figure S6:** Structure prediction and validation of vaccine V1 (A) 3D model (B) Ramachandran layout and (C) The ERRAT quality value (D) Z score graph sequence position (E) Z score graph (number of residues)


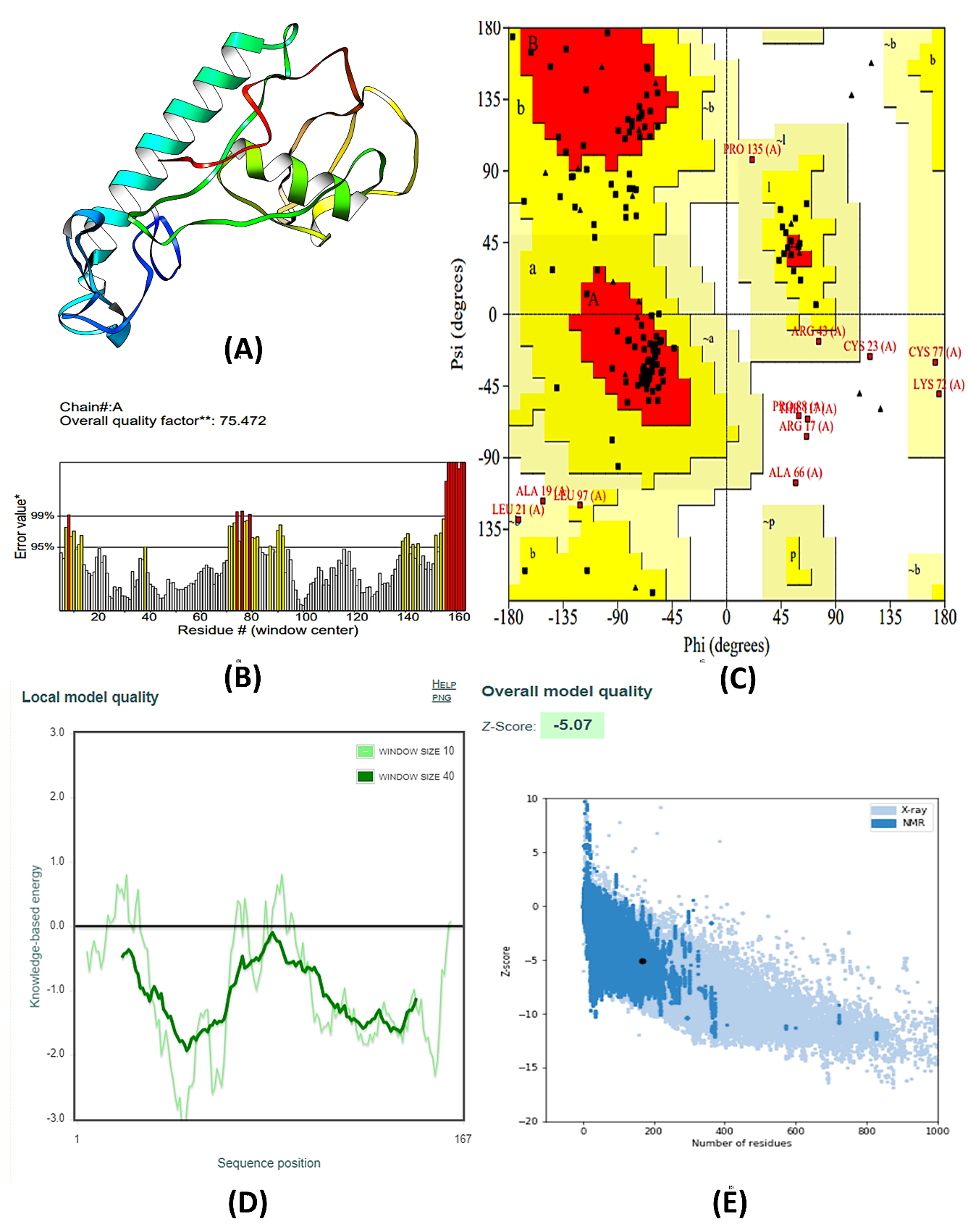


**Supplementary Figure S7:** Structure prediction and validation of vaccine V2 (A) 3D model (B) Ramachandran layout and (C) The ERRAT quality value (D) Z score graph sequence position (E) Z score graph (number of residues)

**(A)**

**(B)**


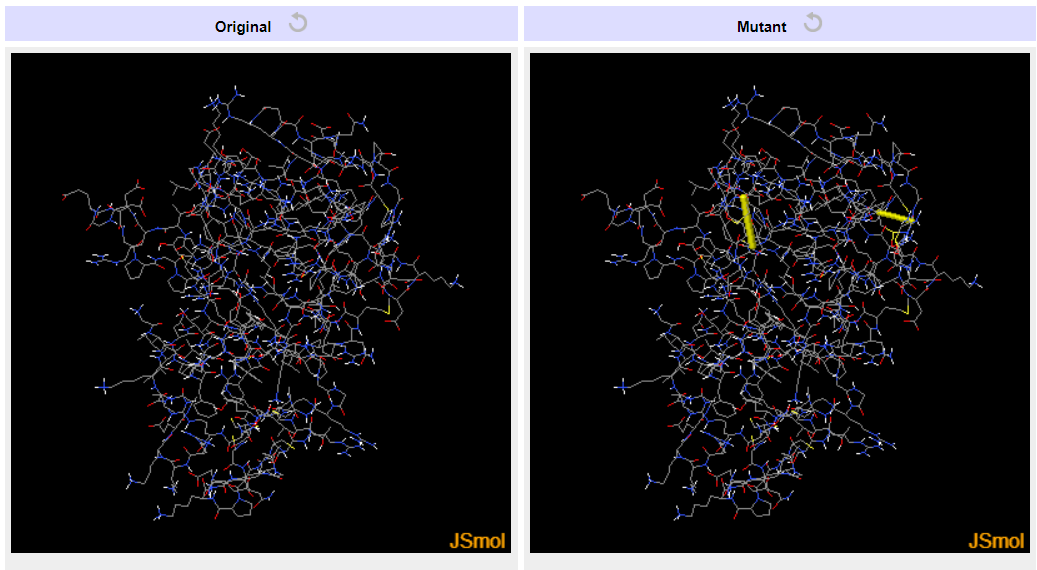


**Supplementary Figure S8:** Disulphide engineering of V1 (A) Original Model without any disulfide bond (B) Mutant Model with two pairs (TRP 74-PRO 161 and ASN 86-GLY 149) that were modified to form disulfide bond.


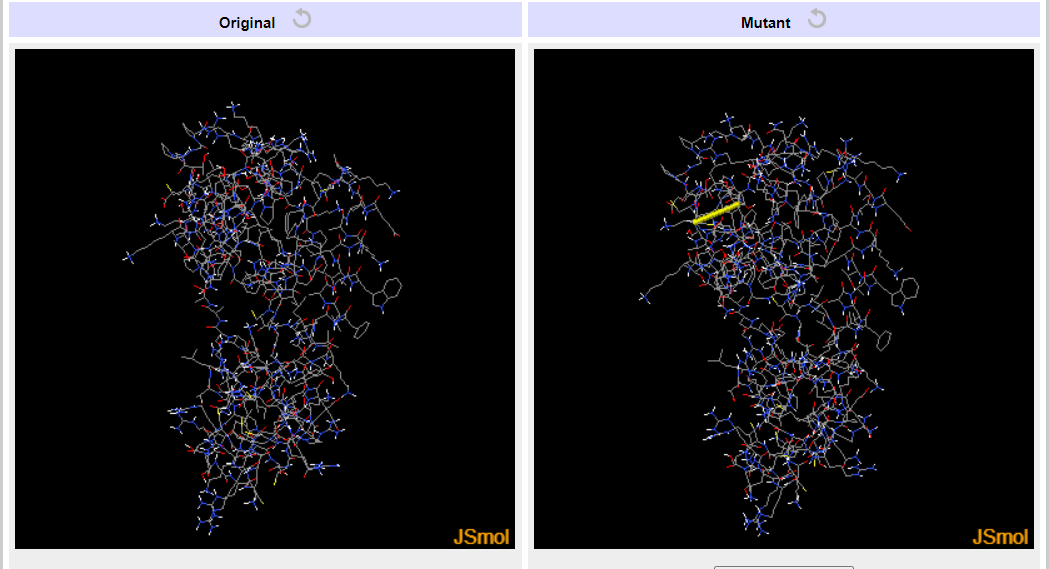


**(B)**

**(A)**

**Supplementary Figure S9:** Disulphide engineering of V1 A) Original Model without any disulfide bond (B) Mutant Model with one pair (ARG 121-GLY 134) that were modified to form disulfide bond.


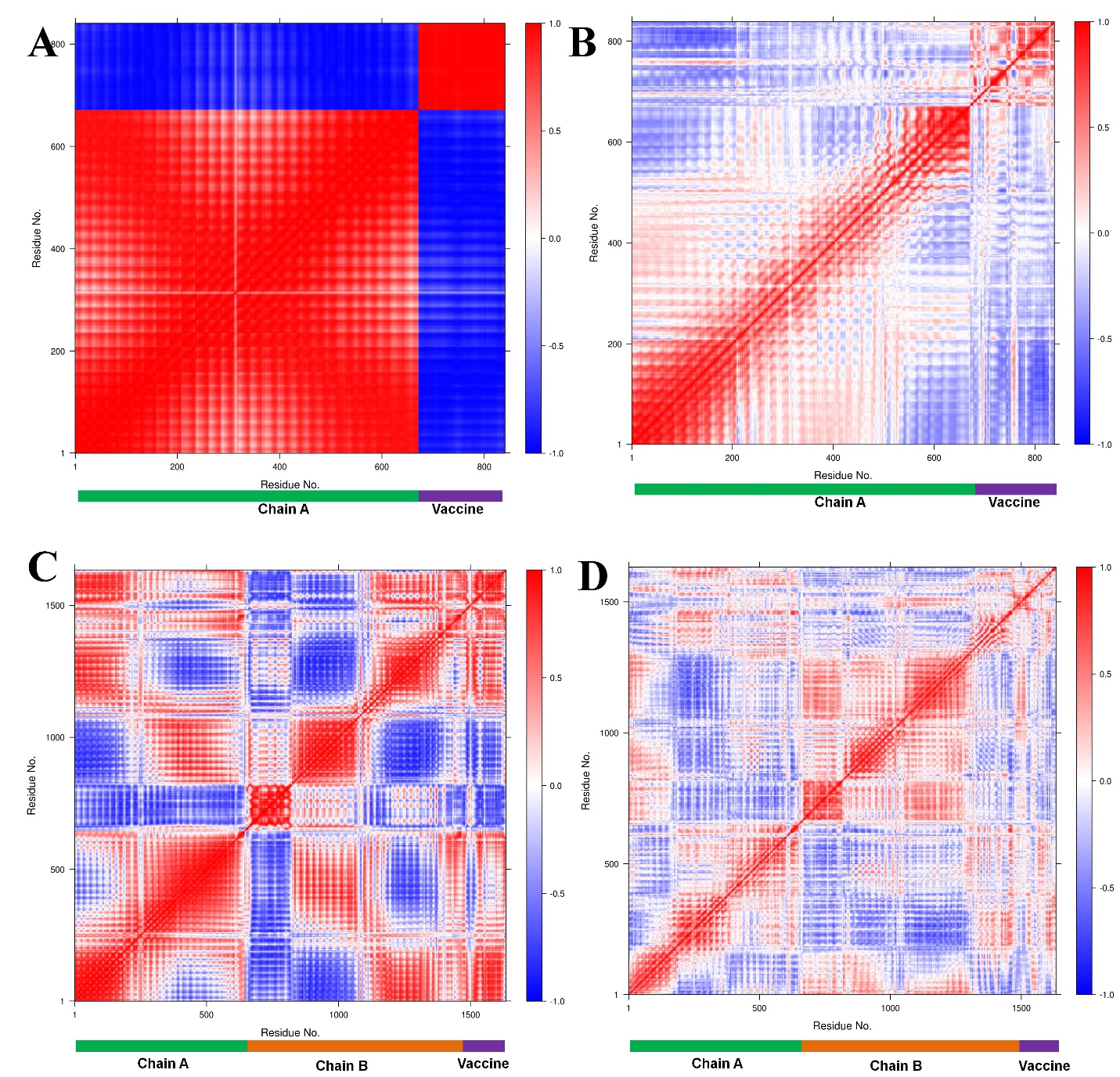


**Supplementary Figure S10:** DCCM analysis. A) TLR3-V1 complex, B) TLR3-V2 complex, C) TLR5-V1 complex, and D) TLR5-V2 complex.


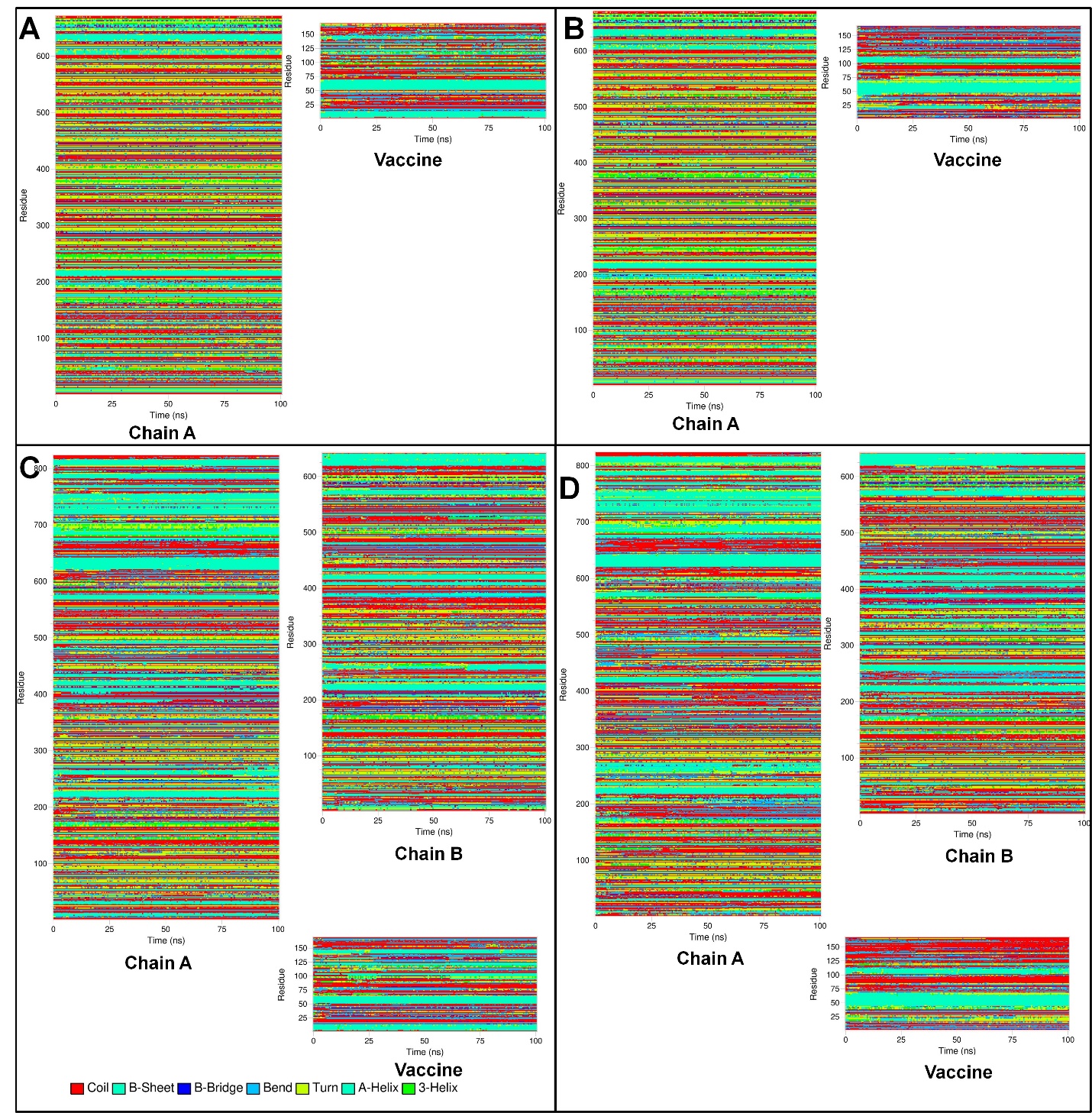


**Supplementary Figure S11:** DSSP plots for individual TLR chains and vaccine constructs. A) TLR3-V1 complex, B) TLR3-V2 complex, C) TLR5-V1 complex, and D) TLR5-V2 complex


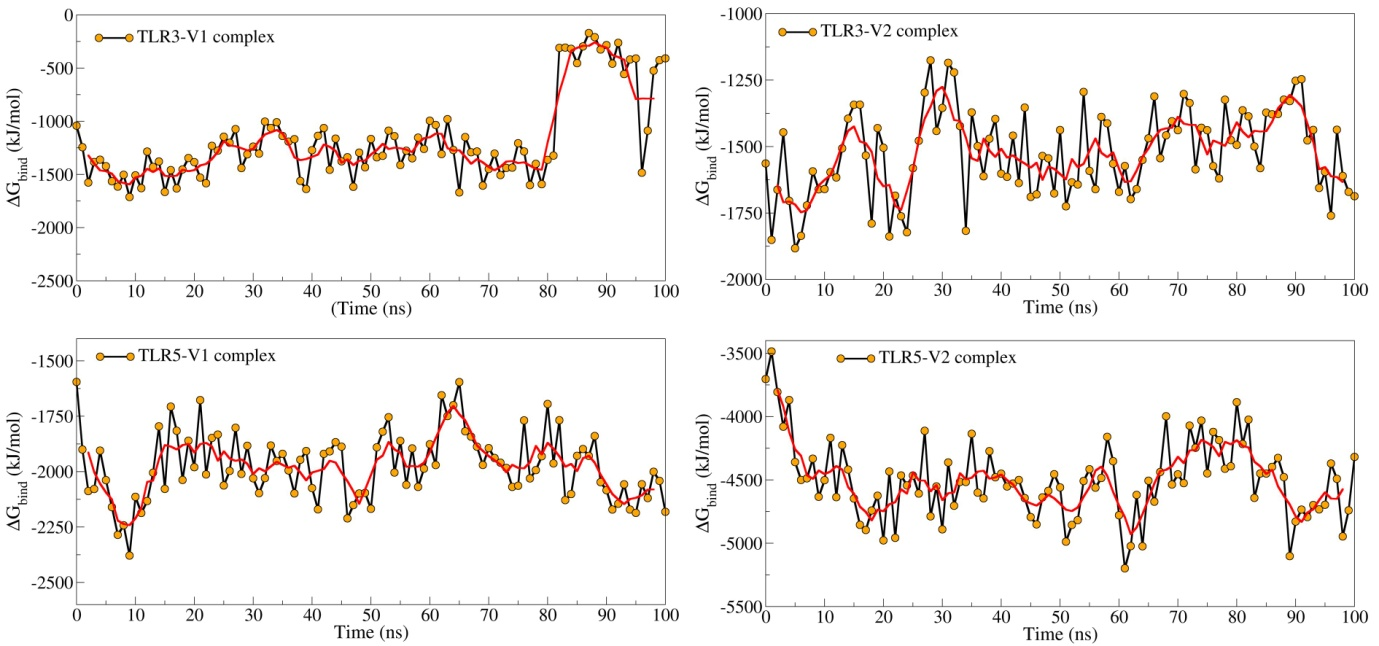


**Supplementary Figure S12:** The plots of MM-PBSA binding energy (ΔGbinding) against simulation time. A) TLR3-V1 complex, B) TLR3-V2 complex, C) TLR5-V1 complex, and D) TLR5-V2 complex.


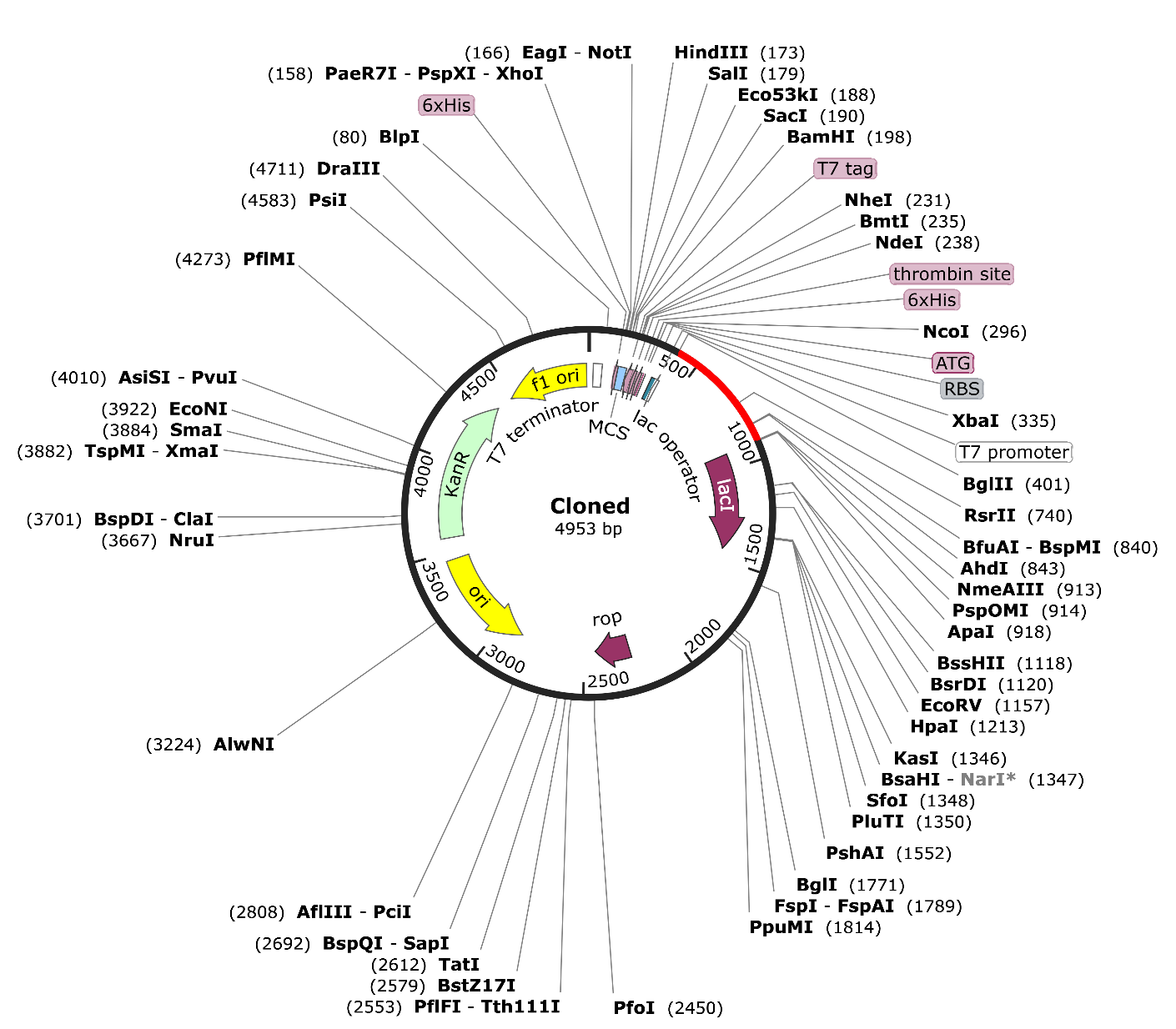


**Supplementary Figure S13:** In silico cloning and codon adaptation of V1


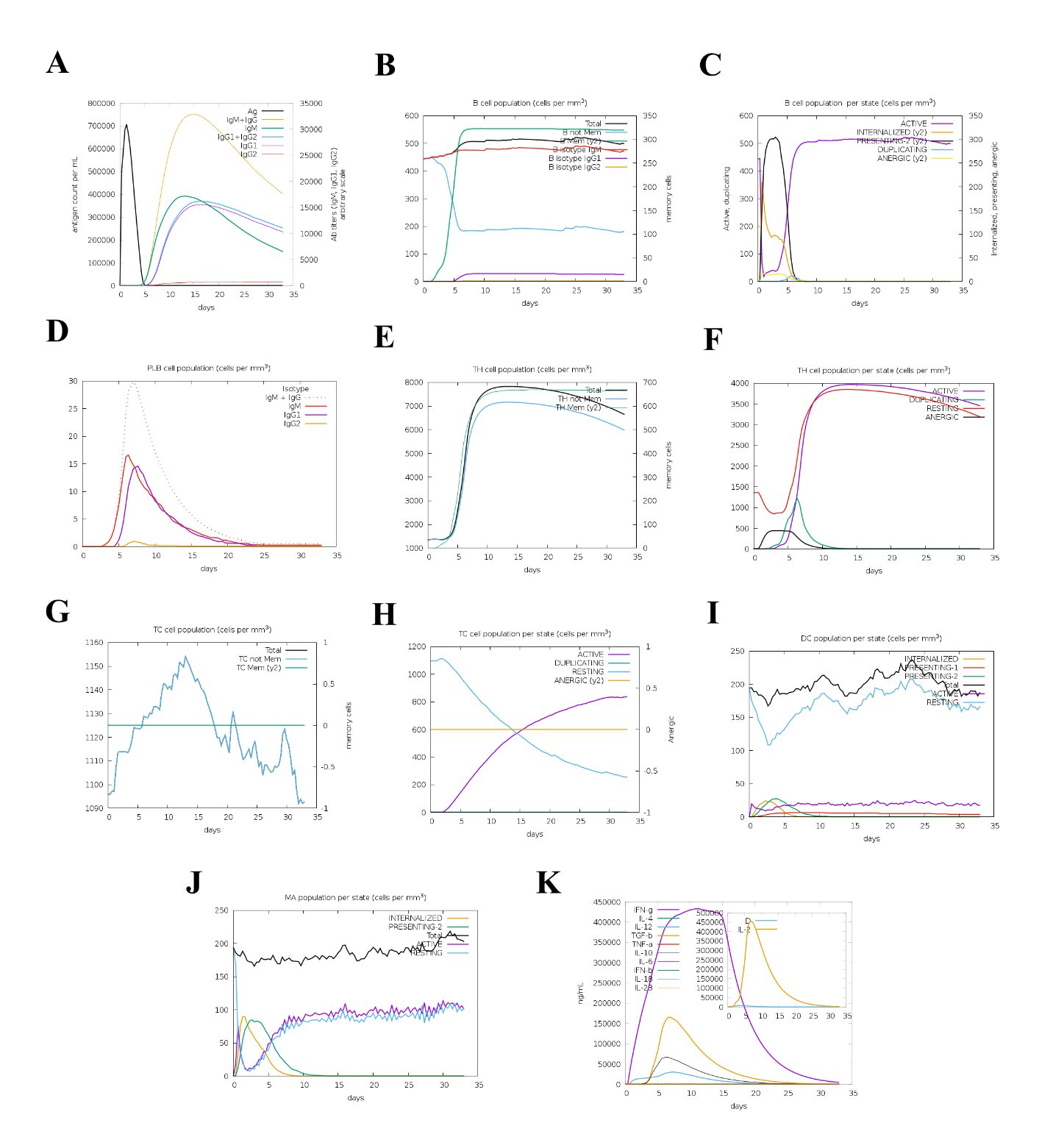


**Supplementary Figure S14:** C-ImmSimm represents the immunological stimulation of the best predicted vaccine. (A) The immunoglobulin and immunocomplex responses to vaccine immunisations (black lines) and the subclasses are delineated by coloured lines. (B) Enhancement in the B-cell population (C) Inclination of the B-cell population by state during immunisation.. (D) Increase in plasma B-cell (PLB) population across the duration of the injections. (E) The helper T-cell (TH) population increased throughout the course of three injections. (F) Enhancement in the helper T-cell population per state throughout vaccination. (G) Enhanced regulatory T lymphocyte (TC) activity throughout the course of three injections. (H) The cytotoxic T lymphocyte population increased during the infusions. (I) Increase in the active cytotoxic T lymphocyte population per state throughout the course of three injections; DC, dendritic cell. (J) Elevation in the active dendritic cell population per state during the three injections; MP and macrophages. (K) The concentrations of several cytokines increased during the course of three doses.
